# Sculpting the Midface and Lower Face: A Novel Biostimulatory Technique Using Hyperdilute Calcium Hydroxylapatite

**DOI:** 10.1093/asjof/ojaf104

**Published:** 2025-08-25

**Authors:** Kalpna Kay Durairaj, Monalea Yambao, Jacob Linnemann-Heath, Arshiya Dhiman

## Abstract

**Background:**

Facial aging is a multifactorial process characterized by soft tissue atrophy, skeletal remodeling, and degradation of dermal extracellular matrix components such as collagen and elastin. Injectable biostimulators have gained popularity in recent years for their efficacy in reinforcing the structural foundation of the face by stimulating the body's natural regenerative processes.

**Objectives:**

The authors of this study aimed to assess their multidepth injection technique with hyperdilute calcium hydroxylapatite (CaHA–CMC; Radiesse, Merz Aesthetics, Raleigh, NC) for multilayered collagen biostimulation to address laxity, jowling, and skin aging-related changes.

**Methods:**

The authors of this study reviewed the charts of 22 patients who underwent 2 treatment sessions with hyperdilute CaHA–CMC (1:3 ratio) using the author's technique for targeted rejuvenation of the mid and lower face. At each visit, assessments included the Global Aesthetic Improvement Scale (GAIS), patient satisfaction, and rankings of aging parameters of the face measured by 5-point photonumeric scales. Volumetric changes were objectively measured with 3-dimensional (3D) imaging analysis via QuantifiCare's 3D Track software.

**Results:**

Quantitative analysis at Day 150 revealed a significant improvement in cheek volume (*P* = .0012) and significant reductions in jowl volume, nasolabial fold depth, and marionette line depth (*P* < .0001, *P* < .0001, *P* = .0019, respectively). GAIS evaluations from the treating physician, a blinded evaluator, and patients demonstrated progressive improvement peaking at Day 150.

**Conclusions:**

The results of this study support the safety and efficacy of the author's novel technique with hyperdilute CaHA for lower and midface rejuvenation, further enhancing the utility of biostimulators in aesthetic medicine.

**Level of Evidence:**

4 (Therapeutic) 
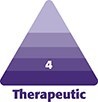

Over the last several years, collagen biostimulators have emerged at the forefront of regenerative aesthetic medicine, gaining traction for their efficacy in establishing the foundational support necessary for sustained soft tissue remodeling and facial rejuvenation. Injectable biostimulators trigger neocollagenesis and fibrillogenesis to stimulate the production of extracellular matrix (ECM) components, such as Collagen type I, Collagen type III, and elastin.^1^ These support the mechanical integrity of the soft tissue envelope of the face.^2^ Although the ECM-stimulating and tissue-strengthening effect of biostimulators has been well established, it has been recently hypothesized that injecting near facial retaining ligaments anchors the surrounding tissues, revitalizing them and creating a mechanical lift of the superficial musculoaponeurotic system (SMAS) and the overlying dermis.^3,4^

Calcium hydroxylapatite–carboxymethylcellulose (CaHA–CMC; Radiesse, Merz Aesthetics, Raleigh, NC) is a biocompatible, resorbable implant product, well established for its capacity for volume replacement as a dermal filler and a biostimulator of multiple components of the ECM.^5-9^ CaHA–CMC consists of 30% smooth CaHA microspheres suspended in a 70% aqueous sodium CMC gel carrier.^10^ Although the soluble gel carrier gradually dissipates, the fibroblasts adhere to the CaHA microspheres, which undergo biodegradation over time, driving the continuous production of collagen and elastin.^11-13^ Light microscopy at 6 months postinjection reveals CaHA microspheres remaining stable at the dermal–subcutaneous junction, surrounded by thick collagen, fibroelastic tissue, and histiocytes, without granuloma formation, migration, or significant inflammation.^13^ Because of its high viscoelasticity, CaHA–CMC provides an immediate 1-to-1 correction upon injection—1 mL of product injected yields ∼1 mL of volume correction.^14^ CaHA–CMC is currently FDA-approved as a subdermal injectable for the treatment of moderate-to-severe facial rhytides and restoration of lipoatrophy among individuals diagnosed with human immunodeficiency virus. CaHA–CMC is also indicated for soft tissue augmentation contour enhancement along the jawline and correction of volume deficiencies in the dorsum of the hands.^15^

In clinical practice, CaHA–CMC is often diluted (1:1) or hyperdiluted (1:>1) with saline and/or lidocaine to partially or entirely diminish the product's direct volumizing effect and increase its spread, thereby focusing on long-term tissue remodeling and enhancing the immediate activation of its biostimulatory properties.^16^ Consequently, diluted and hyperdiluted CaHA–CMC can be used to address tissue laxity and enhance skin quality and tightening—especially across larger facial and body regions—through the stimulation of neocollagenesis, elastogenesis, and angiogenesis.^17-21^ When administered into the subdermal layers of the face, diluted and hyperdiluted CaHA–CMC formulations have been shown to enhance skin tightening while minimizing volumizing effects.^22^ Research indicates that higher dilution ratios facilitate a broader dispersion of CaHA–CMC particles, promoting a more uniform biostimulatory effect across the treated area. The increased particle dispersion allows greater fibroblast interaction with CaHA microspheres, which is hypothesized to stimulate more widespread neocollagenesis.^23^ Because this interaction is dependent on the optimal spacing of the CaHA microspheres, the degree of dilution is a critical factor in maximizing CaHA–CMC's biostimulatory potential.

A 1:1 dilution is frequently used to provide moderate volume enhancement with smoother tissue transitions. A 1:2 dilution, often preferred for facial applications, yields negligible immediate volumization, favoring a pronounced biostimulatory effect.^21,22^ Existing literature suggests that a 1:3 dilution is typically employed for the neck, décolletage, and body, while higher dilutions, such as 1:4, are generally recommended for individuals with thinner skin.^22,24^ However, there is currently no established protocol for using a 1:3 dilution in facial rejuvenation.

To further explore these principles, this retrospective chart review aims to evaluate techniques used in our facial plastic surgery office for collagen biostimulation within the superficial dermis, deep dermis, dermal fat junction, cheek fat pads, and at the supraperiosteal level to address laxity, jowling, and skin aging-related changes. This study assessed the safety and efficacy of a multiple-depth injection technique for mid- and lower-face rejuvenation using hyperdiluted CaHA–CMC (1:3 dilution ratio) for a series of patients followed over 6 months.

## METHODS

This study was conducted as a retrospective cohort study, reviewing charts of patients who presented with mid- and lower-face aging changes and fat pad deflation and underwent treatment for rejuvenation. The review excluded participants who had incomplete treatments or failed to make follow-up visits, participants with piercings or tattoos on the mid and lower face, and participants who have undergone other cosmetic aesthetic treatments in the mid- and lower-face concurrent with the treatment with hyperdilute CaHA–CMC. This review examined charts of patients previously treated with hyperdilute CaHA–CMC in the mid and lower face who completed 2 treatment sessions at D0 and D30 and 2 follow-up visits at D90 and D150. At each treatment session, patients were injected with 1 syringe of CaHA–CMC diluted to a 1:3 ratio. A total of 22 patient charts were reviewed. This study was approved by Allendale Investigational Review Board (KDHDR24), and all study patients provided written informed consent allowing their data to be used for the purpose of this study.

### Treatment Procedure

Each syringe of CaHA–CMC contained 1.5 mL of product. Patients received 1 syringe diluted at a 1:3 ratio per treatment session with 2 treatment sessions, for a total of 2 syringes, given 4 weeks apart. To prepare the dilution, a LuerLock 10 mL syringe containing 0.5 mL of 1% Lidocaine was connected to the CaHA–CMC-containing syringe through a transfer adapter in a sterile mixing environment. At least 20 passes were performed to ensure a homogenous product. The 10 mL syringe containing the mixture was then connected to another 10 mL syringe containing 4.0 mL of normal saline. An additional 20 passes were performed to facilitate homogenization, producing a 1:3 dilution and a total of 6.0 mL of the diluted product.

During each treatment session, topical Lidocaine/Tetracaine 23%/7% ointment cream was applied to the treatment sites. After 15 min of numbing, the topical anesthetic was cleansed off with 70% ethyl alcohol. With the patient sitting in an upright position, the treating physician utilized a fanning/cross-hatching technique with a standard 27 G 0.75-inch needle; 3.0 mL of product was delivered to each side of the face, with 0.75 mL per injection zone. Within each injection zone, approximately one-third of that 0.75 mL (roughly 0.25 mL) was injected into each injection plane: the supraperiosteal plane, the intradermal plane, and within the fat pads (submalar fat compartment, buccal fat compartment, subzygomatic fat compartment, and superior jowl fat compartment). To employ a multilayered approach, treatment was administered in the deep dermis at the dermal fat junction. Injections were performed within the subzygomatic fat pad, the submalar fat pad, the buccal fascial space, and the prejowl sulcus. For the lower face, injections were placed intradermally along marionette lines, feathered toward the mandibular and masseteric ligaments. These intradermal injections were administered in a retrograde linear cross-hatching pattern to address fine lines. Following the procedure, the treatment area was gently massaged with circular motions to facilitate product dispersion. Patients were instructed to continue massaging twice daily for 5 days. A video demonstrating the author's multidepth, multilayered injection technique is available in the Supplemental Digital Content (Video).

### Chart Review

At each visit, standardized photographs of each patient were captured using a Nikon D750 (Tokyo, Japan) and a QuantifiCare Lifeviz Infinity Pro (QuantifiCare Inc., Suwanee, GA). With the Nikon D750, images of a frontal view, 45° oblique view, and profile view were taken of each patient under consistent lighting conditions. Images taken with the Lifeviz Infinity Pro were stitched together in QuantifiCare's 3D Track software to create 3-dimensional (3D) models of each patient. Per standard clinical protocol, the treating physician, and blinded evaluator assessed pertinent characteristics of the mid and lower face at each visit: infraorbital hollowing, upper and lower cheek sunkenness, nasolabial folds, marionette lines, oral commissures, loss of jawline contour, and static and dynamic cheek wrinkles. Each of these aging parameters was defined according to 5-point photonumeric scales, classifying it as none (0), mild (1), moderate (2), severe (3), or very severe (4).

Immediately after each treatment session, patients were asked to report their pain levels according to a numerical rating scale, with 0 indicating no pain and 5 indicating the worst pain they had ever experienced. Patients also rated their satisfaction with the overall treatment experience at each visit using a 5-point Likert scale, ranging from extremely dissatisfied (1) to extremely satisfied (5). At each visit after baseline, patients completed a comprehensive physical exam to evaluate wellness, document any side effects of the treatment, and resolve any concerns. Additionally, the treating physician, blinded evaluator, and the patient rated overall aesthetic improvement using the 5-point Global Aesthetic Improvement Scale (GAIS) at each visit after baseline at D30, D90, and D150.

Using QuantifiCare's 3D Track software, heat maps were created to depict changes in facial volume, based on extrapolated surfaces that respected the natural contours of the face. The 3D Track software quantifies volume variations between this reference surface (extrapolated surface) and the actual surface. Areas with lighter hues trending to red indicate projection beyond the extrapolated surface, whereas darker hues transitioning to blue signify volume deficiencies. This innovative approach to objective visualization delivers key insights into the treatment's impact, revealing the distribution effect of hyperdiluted CaHA–CMC and the subsequent volumization and repositioning of facial tissues.

### Statistical Analysis

Data were represented as the mean ± standard deviation. Ordinal data, such as GAIS, were summarized as the percentage of patients in each category and displayed as stacked bar graphs totaling 100%. The degree of improvement was calculated by averaging the point improvement for each anatomical region. Empirical measurements (eg, cheek volume, jowl volume, NLF depth, and marionette wrinkle depth) were analyzed using paired 2-tailed *t*-tests. Total volume and average fold/wrinkle depths were determined by averaging measurements from the left and right sides of the face at each time point. Statistical analyses were conducted using GraphPad Prism version 10.4.0 (GraphPad Software, Boston, MA). Significance was defined as follows: not significant (ns) *P* > .05, *P < .05, **P < .01, ***P < .001, and ****P < .0001.

## RESULTS

**Figure 1.**
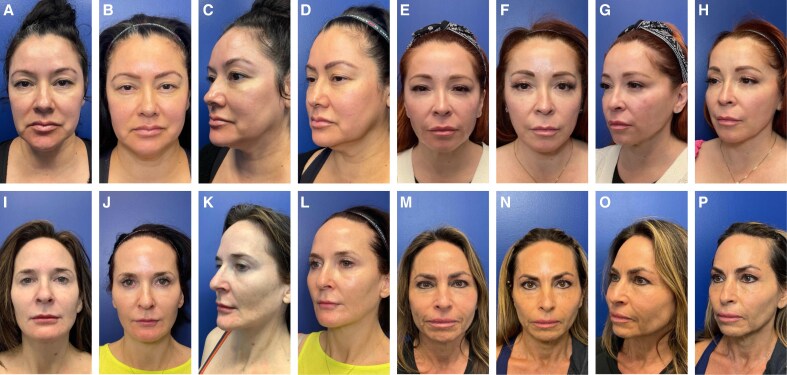
(A) Before and (B) after frontal; (C) before and (D) after oblique views of a 52-year-old female patient. (E) Before and (F) after frontal; (G) before and (H) after oblique views of a 50-year-old female patient. (I) Before and (J) after frontal; (K) before and (L) after oblique views of a 45-year-old female patient. (M) Before and (N) after frontal; (O) before and (P) after oblique views of a 59-year-old female patient.

A total of 22 patient charts were reviewed. The sample consisted of 21 females and 1 male, with a mean age of 51.95 ± 3.94 years and comprising Fitzpatrick skin types I-IV (Table 1). Improvements were observed across all parameters ranked on photonumeric scales (Figure 1).

**Table 1.** Demographic Characteristics of Study Participants (*n* = 22)

Demographic characteristics	Frequency	Percent
**Age**		
<30	0	0%
30-40	2	9.10%
40-50	6	27.30%
50-60	9	40.90%
60-70	4	18.20%
70+	1	4.50%
Gender		
Male	1	4.50%
Female	21	95.50%
Fitzpatrick skin type		
1	2	9.50%
2	5	23.80%
3	10	47.60%
4	4	19.00%
5	0	0%
6	0	0%
Conditions		
Dark spots	7	31.80%
Loose skin	8	36.40%
Wrinkles	11	50.00%
Acne	2	9.10%
Prominent scarring	1	4.50%
Excessive veins	2	9.10%
Tired or droopy eyes	14	63.60%
History of injectable treatments and dermal fillers	5	22.70%
Takes medication	4	18.20%
Previous surgery	5	22.70%

### Global Aesthetic Improvement Scale

**Figure 2.**
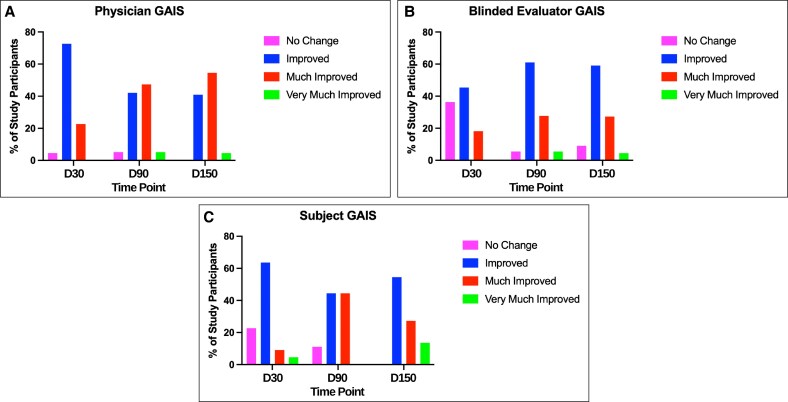
Aesthetic outcomes were validated by improved Global Aesthetic Improvement Scale assessments at D30, D90, and D150 by (A) the treating physician, (B) a blinded evaluator, and (C) the patient.

Overall improvement was also quantitatively measured throughout the treatment period by the treating physician, a blinded evaluator, and the patient using the GAIS. The physician's GAIS assessment revealed that study participants demonstrated a gradual improvement in overall appearance that peaked with 40.9% of patients rated as improved, 54.6% as much improved, and 4.6% as very much improved 6 months after treatment (Figure 2A). A blinded evaluator also noted a similar trend, with progressive improvement peaking at 6 months with 59.1% of patients rated as improved, 27.3% as much improved, and 4.6% as very much improved (Figure 2B). Importantly, positive outcomes were also reflected in the patients' self-evaluations. At D30, 77.3% of patients rated their results as improved, much improved, or very much improved, and by D150, this percentage rose to 95.5% (Figure 2C). GAIS evaluations from the treating physician, evaluator, and patients reflected an overall high degree of progressive improvement peaking at D150.

### Patient Satisfaction and Pain Level

**Figure 3.**
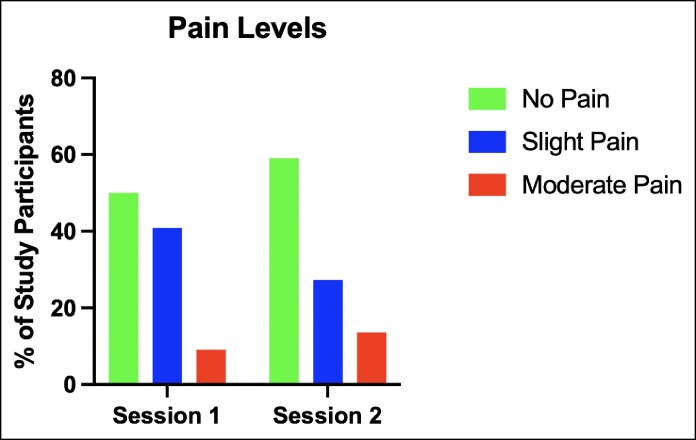
Patients reported treatment pain levels at each treatment session.

Additionally, patient satisfaction with aesthetic appearance and overall treatment, as well as pain scale ratings, was among the secondary endpoints monitored throughout the treatment period. At D150, 72.7% of patients reported extreme satisfaction, and 13.6% reported satisfaction with the treatment. Furthermore, participants experienced minimal discomfort, with no patients reporting severe or extreme pain, and 9.1% and 13.6% reporting moderate pain at treatment Sessions 1 and 2, respectively (Figure 3). Strong patient satisfaction is further supported by the treatment's favorable safety profile, as no serious complications were reported during the treatment period. One patient experienced swelling, which resolved completely within ∼4 weeks with a short course of anti-inflammatory oral steroids.

### QuantifiCare's Objective Measurements

**Figure 4.**
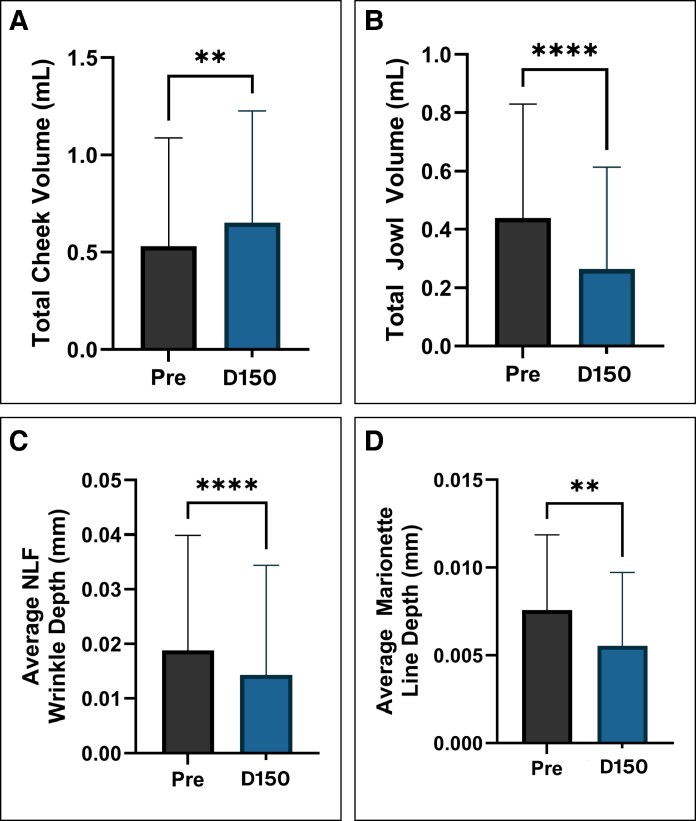
Mean volumetric measurements demonstrate significant improvements at D150. (A) Mean cheek volume measurements demonstrate significant improvement (paired *t*-test, 2-sided, **P < .01). (B) Mean jowl volume measurements demonstrate significant reduction (paired *t*-test, 2-sided, ****P < .0001). (C) Mean nasolabial fold depth measurements demonstrate significant reduction (paired *t*-test, 2-sided, ****P < .0001). (D) Mean marionette line depth measurements demonstrate significant reduction (paired *t*-test, 2-sided, **P < .01).

**Figure 5.**
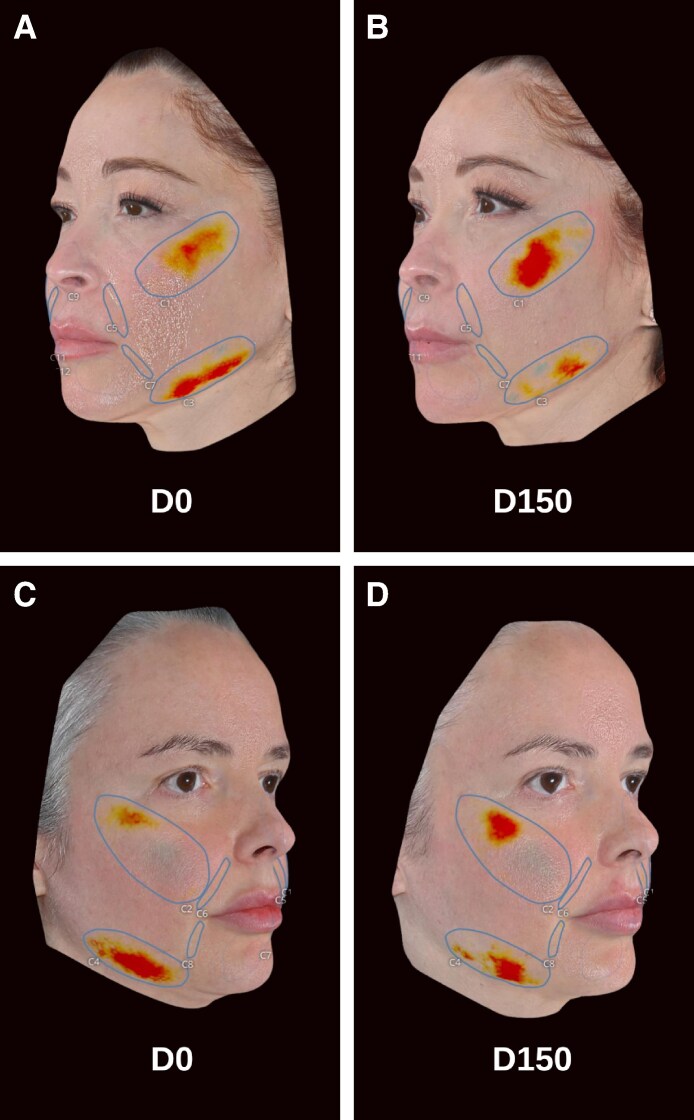
Defined contours in QuantifiCare's 3D Track software demonstrating elevated cheek projection (expansion of red hues) and a reduction in jowl heaviness (shrinkage of red hues) at D150 with the author's hyperdilute CaHA technique. (A, B) Facial scans from a 50-year-old female study participant. (C, D) Facial scans from a 39-year-old female study participant.

QuantifiCare's 3D imaging analysis was used to measure changes in cheek volume, jowl volume, nasolabial fold depth, and marionette line depth (Table 2). Comparing baseline scans to D150 scans, significant increases in mean cheek volume were displayed (*P* = .0012; Figure 4A). However, improvement varied between sides, with the left side demonstrating an average increase of 32.87% (*P* = .0022) and the right side showing an average increase of 15.10% (*P* = .1788; Supplemental Figure 1A, B). Patients exhibited significant reductions in jowl volume, with a mean reduction of 0.087 mL in jowl volume by D150, corresponding to a 40.82% decrease from baseline (*P* = <.0001; Figure 4B). Volumetric analysis demonstrated a 36.46% reduction in jowl volume on the left side of the face and a 45.17% reduction on the right side (*P* = .0008, *P* ≤ .0001, respectively; Supplemental Figure 2A, B). Significant reductions in nasolabial fold depth were also measured, with a mean improvement of 27.88% (*P* ≤ .0001; Figure 4C). Decreases in nasolabial fold depth varied between sides, with the left side showing an average improvement of 42.14% and the right side showing an average improvement of 13.61% (*P* = .0051, *P* = .0444, respectively; Supplemental Figure 3A, B). The depth of the marionette line reflected a 26.94% mean reduction from baseline (*P* = .0019; Figure 4D). Volumetric analysis demonstrated a 30.57% reduction in marionette line depth on the left side of the face and a 23.30% reduction on the right side (*P* = .0032, *P* = .0476, respectively; Supplemental Figure 4A, B). QuantifiCare's 3D imaging heat maps comparing baseline scans to D150 scans consistently revealed an increase in cheek volumization (expansion of red hues) and a reduction in jowl volume (shrinkage of red hues; Figure 5).

**Table 2.** Improvements Calculated by QuantifiCare's 3D Track Software

Volumetric measurements	Average at baseline	Average at D150	Percent change	*P*-value
Left cheek volume (mL)	0.2331 ± 0.1151	0.3098 ± 0.1362	+32.87	.0022
Right cheek volume (mL)	0.2974 ± 0.1561	0.3423 ± 0.1383	+15.10	.1788
Left jowl volume (mL)	0.2705 ± 0.1087	0.1719 ± 0.0876	−36.46	.0008
Right jowl volume (mL)	0.1680 ± 0.1000	0.0921 ± 0.0864	−45.17	<.0001
Left NLF depth (mm)	0.0136 ± 0.0051	0.0079 ± 0.0023	−42.14	.0051
Right NLF depth (mm)	0.0240 ± 0.0157	0.0208 ± 0.0171	−13.61	.0444
Left marionette line depth (mm)	0.0071 ± 0.0024	0.0050 ± 0.0023	−30.57	.0032
Right marionette line depth (mm)	0.0080 ± 0.0029	0.0061 ± 0.0026	−23.30	.0476

### Facial Characteristics According to Photonumeric Scales

**Figure 6.**
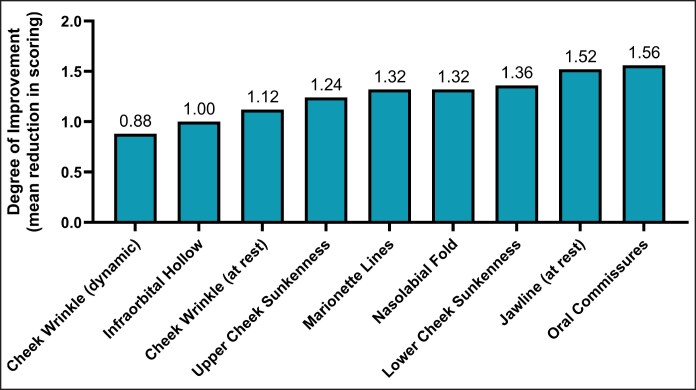
Average improvement over 6 months in facial characteristic rankings of the mid and lower face based on photonumeric scales. Assessments were performed by the treating physician.

Facial rankings of aging characteristics of the mid and lower face—including deep infraorbital hollowing, upper and lower cheek sunkenness, deep nasolabial folds and marionette lines, sagging oral commissures and jawline contour, and static and dynamic cheek wrinkles—reflected collective improvement (Figure 6). Infraorbital hollowing indicated a mean improvement in scoring by 1.00 point on the photonumeric scale. One hundred and fifty days posttreatment, 15.9% of patients improved to no hollowing, 47.7% displayed mild hollowing (up from 31.8% at baseline), 34.1% displayed moderate hollowing (down from 38.6%), and only 2.3% displayed severe infraorbital hollowing (down from 27.3% at baseline; Supplemental Figure 5A).

Upper cheek sunkenness indicated a mean improvement in scoring by 1.24 points on the photonumeric scale. At baseline, 72.7% of patients displayed moderate to very severe upper cheek sunkenness. One hundred and fifty days posttreatment, this proportion of patients decreased to 22.7%, and 31.8% displayed a full upper cheek (Supplemental Figure 5B). Lower cheek sunkenness indicated a mean improvement in scoring by 1.36 points on the photonumeric scale. At baseline, 81.8% of patients displayed moderate to very severe lower cheek sunkenness. One hundred and fifty days posttreatment, this percentage dropped to 20.5%, with a notable shift toward milder or no sunkenness—43.2% exhibited mild sunkenness and 36.4% exhibited a full lower cheek (Supplemental Figure 5C).

Nasolabial folds indicated a mean improvement in scoring by 1.32 points on the photonumeric scale. One hundred and fifty days posttreatment, cases with severely deep nasolabial folds dropped from 34.1% to 6.8%, moderate cases rose from 40.9% to 45.5%, mild cases rose from 20.5% to 40.9%, and 6.8% exhibited no folds (Supplemental Figure 5D). Similarly, marionette lines indicated a mean improvement in scoring by 1.32 points on the photonumeric scale. One hundred and fifty days posttreatment, cases with severely deep marionette lines improved from 40.9% to 11.4%, moderate cases rose from 25% to 45.5%, mild cases improved from 15.9% to 34.1%, and 4.6% of patients presented with no folds (Supplemental Figure 5E).

Oral commissures indicated a mean improvement in scoring by 1.56 points on the photonumeric scale. One hundred and fifty days posttreatment, patients presenting with very severe downturns of the mouth improved from 25% to 2.3%, 27.3% presented with severe downturns (up from 25%), and 18.2% presented with moderate downturns (down from 25%). There was a substantial transition toward milder or absent downturns, with mild cases increasing from 25% to 34.1%, and 18.2% of patients exhibiting no downturns (Supplemental Figure 5F). Jawline contour at rest indicated a mean improvement in scoring by 1.52 points on the photonumeric scale. At baseline, 86.4% of patients displayed moderate to very severe sagging. One hundred and fifty days posttreatment, this proportion dropped to 36.4%, mild cases rose to 43.2%, and 20.5% displayed no sagging (Supplemental Figure 5G).

To assess dynamic cheek wrinkles, patients were asked to smile repeatedly. Dynamic cheek wrinkles indicated a mean improvement in scoring by 0.88 points on the photonumeric scale. Before treatment, 52.3% of patients displayed moderate to very severe wrinkling. One hundred and fifty days posttreatment, this percentage dropped to 22.7%, the proportion of mild cases rose to 68.2%, and 9.1% displayed no wrinkling (Supplemental Figure 5H). Cheek wrinkles at rest indicated a mean improvement in scoring by 1.12 points on the photonumeric scale. Initially, 70.5% of patients had moderate to very severe wrinkling. One hundred and fifty days posttreatment, this percentage dropped to 25.0%, with 63.6% displaying mild wrinkling and 11.4% displaying no wrinkling (Supplemental Figure 5I).

## DISCUSSION

Facial aging is a multidimensional process influenced by changes at the skeletal, subcutaneous, and dermal levels. The mid and lower faces are particularly susceptible to the gradual depletion of collagen, leading to clinically evident signs of aging, such as skin laxity, rhytids, and gravitational drooping. Biostimulatory injectables, such as CaHA–CMC, have become one of the cornerstones of modern aesthetics, gaining traction for their ability to address aging-related changes while building a strong foundation for long-term maintenance. This study provides valuable insights into the safety and efficacy of hyperdiluted CaHA–CMC for mid- and lower-face rejuvenation using the author's multidepth, multilayered injection technique.

Facial subcutaneous fat is highly compartmentalized, with ligaments often forming where fat compartments meet.^25^ Injecting collagen biostimulators near the zygomatic ligament provides structural reinforcement that can support and elevate the soft tissues of the mid and lower face, leading to a more youthful appearance. By augmenting ligament strength in this region, the midface malar fat pads can be repositioned, counteracting the natural descent of facial tissues that occurs with aging. This redistribution of support exerts a vector of upward tension, reducing the prominence of jowling and diminishing the appearance of nasolabial folds and marionette lines. Consequently, tone is improved in the lower face, as laxity and lower facial heaviness are improved. CaHA–CMC's biostimulatory properties contribute to longer-term tissue remodeling, which should help to maintain and reinforce this lifted position.

This research presents an innovative demonstration that a minimal number of syringes can adequately treat both sides of the face in multiple zones to create a global facial optimization effect. Collagen has a half-life of ∼14.8 years based on aspartic acid racemization rates.^26^ In addition to stimulating neocollagenesis and neoelastinogenesis, CaHA–CMC promotes the synthesis of other essential ECM components, including proteoglycans and glycosaminoglycans, further supporting structural integrity and hydration of the dermal matrix to create an environment that supports overall regenerative success.^7^ Recent histological evidence suggests that biostimulatory response to hyperdiluted CaHA–CMC is not significantly correlated with age within the 39 to 62 year age bracket, further supporting the suitability of this treatment across a broad adult population.^27^ The bioregenerative mechanisms triggered via this technique, particularly the production of long-lasting collagen, suggest the potential persistence of rejuvenating effects beyond the timeframe of this study, positioning it as a valuable option for aesthetic patients seeking a bioregenerative approach to aging.

The findings reaffirm that the therapeutic outcomes of hyperdiluted CaHA–CMC rely on CaHA particle distribution, appropriate dilution, and rationalization of injection location, depth, and plane. By diluting CaHA–CMC to a 1:3 ratio, this study aimed to achieve a particle dispersion that facilitated more widespread fibroblast interaction rather than direct volumization. This approach involving only 2 syringes (3 mL undiluted product total) injected using a multidepth, multiplanar technique yielded comparable aesthetic outcomes to those reported in hyaluronic acid–based full-face rejuvenation studies using significantly higher volumes with an average range of 4.7 to 6.7 mL.^28-30^ Although the relative contributions of the hyperdilution cannot be definitively separated based on this study design, the results underscore the potential of this approach. Quantitative improvements were observed across all measured areas, including reductions in infraorbital hollowing, marionette lines, and nasolabial folds. Additionally, the objective data from 3D imaging analysis revealed notable midface volume restoration and skin tightening effects, emphasizing CaHA–CMC's efficacy in addressing facial tissue laxity and volume loss.

High patient satisfaction scores and minimal adverse effects further highlight the potential of this treatment modality. The GAIS demonstrated substantial aesthetic improvements, with the majority of patients reporting an increasing degree of improvement as the study period progressed. The favorable safety profile, with no reported adverse events, supports the utility of hyperdiluted CaHA–CMC as a minimally invasive option for facial rejuvenation.

The observed difference in treatment outcomes between the right and left sides of the face following CaHA–CMC injections may be partially attributed to natural asymmetries in facial structure. An observational study involving 315 patients has demonstrated that the right side of the face tends to be slightly larger than the left, which could influence how biostimulatory agents distribute and interact with tissue.^31^ A greater surface area or volume on the right side may require a higher product dose to achieve results comparable with the left side. Additionally, differences in mechanical strain from habitual facial expressions and dominant-sided muscle activity could further affect tissue remodeling, potentially leading to a less pronounced or delayed response on the right side. However, this discrepancy could also be attributed to natural facial asymmetry or external factors such as sleeping position, as chronic pressure from 1-sided sleeping may influence circulation, lymphatic drainage, and overall tissue response to treatment.

### Limitations

Further exploration of the concept of multiple-depth collagen biostimulatory treatments is warranted to refine this method and optimize outcomes for skin tightening measures. Despite promising results, the study's retrospective nature and smaller sample size limit the generalizability of its findings. The lack of a control group and the absence of objective measures for collagen density (eg, histological analysis) constrain the ability to fully correlate the biostimulatory effects with the patients' aesthetic outcomes. Although GAIS assessments are inherently subjective, an independent, blinded evaluator was incorporated to provide objective assessments and minimize any potential bias. Modifications in injection zones, depths, techniques, and varying dilution ratios to determine optimal neocollagenesis may yield even greater efficacy. Ideal dilution ratios may vary depending on the anatomical region, individual tissue characteristics, and specific treatment goals, so further investigation is necessary to optimize dilution volumes to create maximum collagen stimulation for different clinical applications. Additionally, incorporating longer follow-up periods could provide insights into the longevity of collagen stimulation and clinical outcomes. Future research may explore innovative approaches to further bolster facial suspensory ligaments and SMAS support of the face, new injection techniques (such as this multidepth multiplanar approach involving supraperiosteal stimulation, deep dermal, and facial fat pad support), and the combination of micronutrient therapies to unlock additional benefits.

## CONCLUSIONS

In conclusion, the authors demonstrate that hyperdiluted CaHA–CMC is a safe and effective option for mid and lower face rejuvenation, offering significant aesthetic improvements and high patient satisfaction. The bioregenerative treatment with hyperdiluted CaHA–CMC resulted in substantial improvements in skin quality and facial volumization, as supported through patient testimonials and improved GAIS ratings and validated with 3D photography. In this study, the authors highlight sustained impact and potential of hyperdilute CaHA–CMC treatments in addressing the complex challenges associated with facial aging and volume changes, further enhancing the utility of biostimulators in aesthetic medicine. This retrospective review of the author's technique supports the potential benefits of hyperdiluted CaHA–CMC in promoting neocollagenesis and improving skin quality, with aesthetic enhancements noted across several parameters.

## Supplemental Material

This article contains supplemental material located online at https://doi.org/10.1093/asjof/ojaf104.

## Supplementary Material

ojaf104_Supplementary_Data

## References

[1] Yutskovskaya Y, Kogan E, Leshunov E. A randomized, split-face, histomorphologic study comparing a volumetric calcium hydroxylapatite and a hyaluronic acid-based dermal filler. J Drugs Dermatol. 2014;13:1047–1052. https://jddonline.com/articles/a-randomized-split-face-histomorphologic-study-comparing-a-volumetric-calcium-hydroxylapatite-and-a-S1545961614P1047X/25226004

[2] Casabona G, Alfertshofer M, Kaye K, et al Ex vivo product distribution of injectable biostimulator substances. Aesthet Surg J. 2023;43:NP348–NP356. doi: 10.1093/asj/sjad014 36662772

[3] Brandt MG, Hassa A, Roth K, Wehrli B, Moore CC. Biomechanical properties of the facial retaining ligaments. Arch Facial Plast Surg. 2012;14:289–294. doi: 10.1001/archfacial.2011.1533 22351846

[4] Cong L-Y, Duan J, Luo C-E, Luo S-K. Injectable filler technique for face lifting based on dissection of true facial ligaments. Aesthet Surg J. 2021;41:NP1571–NP1583. doi: 10.1093/asj/sjaa348 33300562

[5] Botsali A, Erbil H, Eşme P, Gamsızkan M, Aksoy AO, Caliskan E. The comparative dermal stimulation potential of constant-volume and constant-amount diluted calcium hydroxylapatite injections versus the concentrated form. Dermatol Surg. 2023;49:871–876. doi: 10.1097/DSS.0000000000003874 37399137

[6] Silvers SL, Eviatar JA, Echavez MI, Pappas AL. Prospective, open-label, 18-month trial of calcium hydroxylapatite (Radiesse) for facial soft-tissue augmentation in patients with human immunodeficiency virus-associated lipoatrophy: one-year durability. Plast Reconstr Surg. 2006;118:34S–45S. doi: 10.1097/01.prs.0000234847.36020.52 16936543

[7] Zerbinati N, D’Este E, Parodi PC, Calligaro A. Microscopic and ultrastructural evidences in human skin following calcium hydroxylapatite filler treatment. Arch Dermatol Res. 2017;309:389–396. doi: 10.1007/s00403-017-1734-3 28324170 PMC5486564

[8] González N, Goldberg DJ. Evaluating the effects of injected calcium hydroxylapatite on changes in human skin elastin and proteoglycan formation. Dermatol Surg. 2019;45:547–551. doi: 10.1097/DSS.0000000000001809 30893178

[9] Hu Y, Lu H, Yuan X, Yang Z, Gao Q, Qi Z. The histologic reaction and permanence of hyaluronic acid gel, calcium hydroxylapatite microspheres, and extracellular matrix bio gel. J Cosmet Dermatol. 2023;22:2685–2691. doi: 10.1111/jocd.15767 37082836

[10] McCarthy AD, Hartmann C, Durkin A, Shahriar S, Khalifian S, Xie J. A morphological analysis of calcium hydroxylapatite and poly-l-lactic acid biostimulator particles. Skin Res Technol. 2024;30:e13764. doi: 10.1111/srt.13764 38853456 PMC11163027

[11] Aguilera SB, McCarthy A, Khalifian S, Lorenc ZP, Goldie K, Chernoff WG. The role of calcium hydroxylapatite (Radiesse) as a regenerative aesthetic treatment: a narrative review. Aesthet Surg J. 2023;43:1063–1090. doi: 10.1093/asj/sjad173 37635437 PMC11025388

[12] Berlin AL, Hussain M, Goldberg DJ. Calcium hydroxylapatite filler for facial rejuvenation: a histologic and immunohistochemical analysis. Dermatol Surg. 2008;34:S64–S67. doi: 10.1111/j.1524-4725.2008.34245.x 18547184

[13] Marmur ES, Phelps R, Goldberg DJ. Clinical, histologic and electron microscopic findings after injection of a calcium hydroxylapatite filler. J Cosmet Laser Ther. 2004;6:223–226. doi: 10.1080/147641704100003048 16020207

[14] Guida S, Galadari H. A systematic review of Radiesse/calcium hydroxylapatite and carboxymethylcellulose: evidence and recommendations for treatment of the face. Int J Dermatol. 2024;63:150–160. doi: 10.1111/ijd.16888 37897174

[15] Instructions for Use—Radiesse Injectables. Radiesse Injectables. Published July 2024. Accessed January 3, 2025. https://radiesse.com/instructions-for-use/.

[16] McCarthy AD, Soares DJ, Chandawarkar A, El-Banna R, Hagedorn N. Dilutional rheology of Radiesse: implications for regeneration and vascular safety. J Cosmet Dermatol. 2024;23:1973–1984. doi: 10.1111/jocd.16216 38357772

[17] Massidda E. Starting point for protocols on the use of hyperdiluted calcium hydroxylapatite (Radiesse®) for optimizing age-related biostimulation and rejuvenation of face, neck, décolletage and hands: a case series report. Clin Cosmet Investig Dermatol. 2023;16:3427–3439. doi: 10.2147/CCID.S420068 PMC10693750 38050476

[18] de Almeida AT, Figueredo V, da Cunha ALG, et al Consensus recommendations for the use of hyperdiluted calcium hydroxyapatite (Radiesse) as a face and body biostimulatory agent. Plast Reconstr Surg Glob Open. 2019;7:e2160. doi: 10.1097/GOX.0000000000002160 31044123 PMC6467620

[19] Zerbinati N, Calligaro A. Calcium hydroxylapatite treatment of human skin: evidence of collagen turnover through picrosirius red staining and circularly polarized microscopy. Clin Cosmet Investig Dermatol. 2018;11:29–35. doi: 10.2147/CCID.S143015 PMC5772396 29391818

[20] Doyle A, Looi I, Chu P. Microfocused ultrasound with visualization (MFU-V) and hyperdilute calcium hydroxylapatite (CaHA-CMC) of the lower face and submentum to treat skin laxity: a pilot study demonstrating superiority of MFU-V first followed by hyperdilute CaHA-CMC. Aesthet Surg J. 2025;45:305–312. doi: 10.1093/asj/sjae226 39511699

[21] Rovatti PP, Pellacani G, Guida S. Hyperdiluted calcium hydroxylapatite 1:2 for mid and lower facial skin rejuvenation: efficacy and safety. Dermatol Surg. 2020;46:e112–e117. doi: 10.1097/DSS.0000000000002375 32205749

[22] Goldie K, Peeters W, Alghoul M, et al Global consensus guidelines for the injection of diluted and hyperdiluted calcium hydroxylapatite for skin tightening [published correction appears in Dermatol Surg. 2019 Feb;45(2):327. doi: 10.1097/DSS.0000000000001782.]. Dermatol Surg. 2018;44:S32–S41. doi: 10.1097/DSS.0000000000001685 30358631

[23] Nowag B, Casabona G, Kippenberger S, Zöller N, Hengl T. Calcium hydroxylapatite microspheres activate fibroblasts through direct contact to stimulate neocollagenesis. J Cosmet Dermatol. 2023;22:426–432. doi: 10.1111/jocd.15521 36575882

[24] Lorenc ZP, Black JM, Cheung JS, et al Skin tightening with hyperdilute CaHA: dilution practices and practical guidance for clinical practice. Aesthet Surg J. 2022;42:NP29–NP37. doi: 10.1093/asj/sjab269 34192299 PMC8849118

[25] Rohrich RJ, Pessa JE. The fat compartments of the face: anatomy and clinical implications for cosmetic surgery. Plast Reconstr Surg. 2007;119:2219–2227. doi: 10.1097/01.prs.0000265403.66886.54 17519724

[26] Verzijl N, DeGroot J, Thorpe SR, et al Effect of collagen turnover on the accumulation of advanced glycation end products. J Biol Chem. 2000;275:39027–39031. doi: 10.1074/jbc.M006700200 10976109

[27] Doyle A, Looi I, Chu P, Martinez KA, McCarthy AD. Exploratory analysis of age-related trends in biostimulatory response to combined CaHA-CMC and MFU-V treatments. Aesthet Surg J Open Forum. 2025;7:ojaf023. doi: 10.1093/asjof/ojaf023 40351556 PMC12062575

[28] Dhillon B, Patel T. A retrospective analysis of full-face dermal filler treatments: product choice, volume use, and treatment locations. J Clin Aesthet Dermatol. 2020;13:33–40. https://pmc.ncbi.nlm.nih.gov/articles/PMC7577331/PMC7577331 33133339

[29] Rzany B, Cartier H, Kestemont P, et al Full-face rejuvenation using a range of hyaluronic acid fillers: efficacy, safety, and patient satisfaction over 6 months. Dermatol Surg. 2012;38:1153–1161. doi: 10.1111/j.1524-4725.2012.02470.x 22759252

[30] Saliani MT. Full-face rejuvenation with hyaluronic acid fillers based on the MD codes technique: a retrospective, single-center study. J Clin Exp Dermatol Res. 2020;11:1–6. https://www.longdom.org/abstract/fullface-rejuvenation-with-hyaluronic-acid-fillers-based-on-the-md-codes-technique-a-retrospective-singlecenter-study-54445.html

[31] Talisman R, Arnon O, Weinberger A. Facial asymmetry, the right-side dominance: a retrospective analysis of 315 consecutive series of patients. JPRAS Open. 2022;35:18–23. doi: 10.1016/j.jpra.2022.11.001 36593865 PMC9804005

