# Supplementary figures and images for: Sculpting the Midface and Lower Face: A Novel Biostimulatory Technique Using Hyperdilute Calcium Hydroxylapatite
Source: Aesthet Surg J Open Forum. 2025 Aug 25;7:ojaf104. doi: 10.1093/asjof/ojaf104 (PMC12686807; doi:10.1093/asjof/ojaf104)

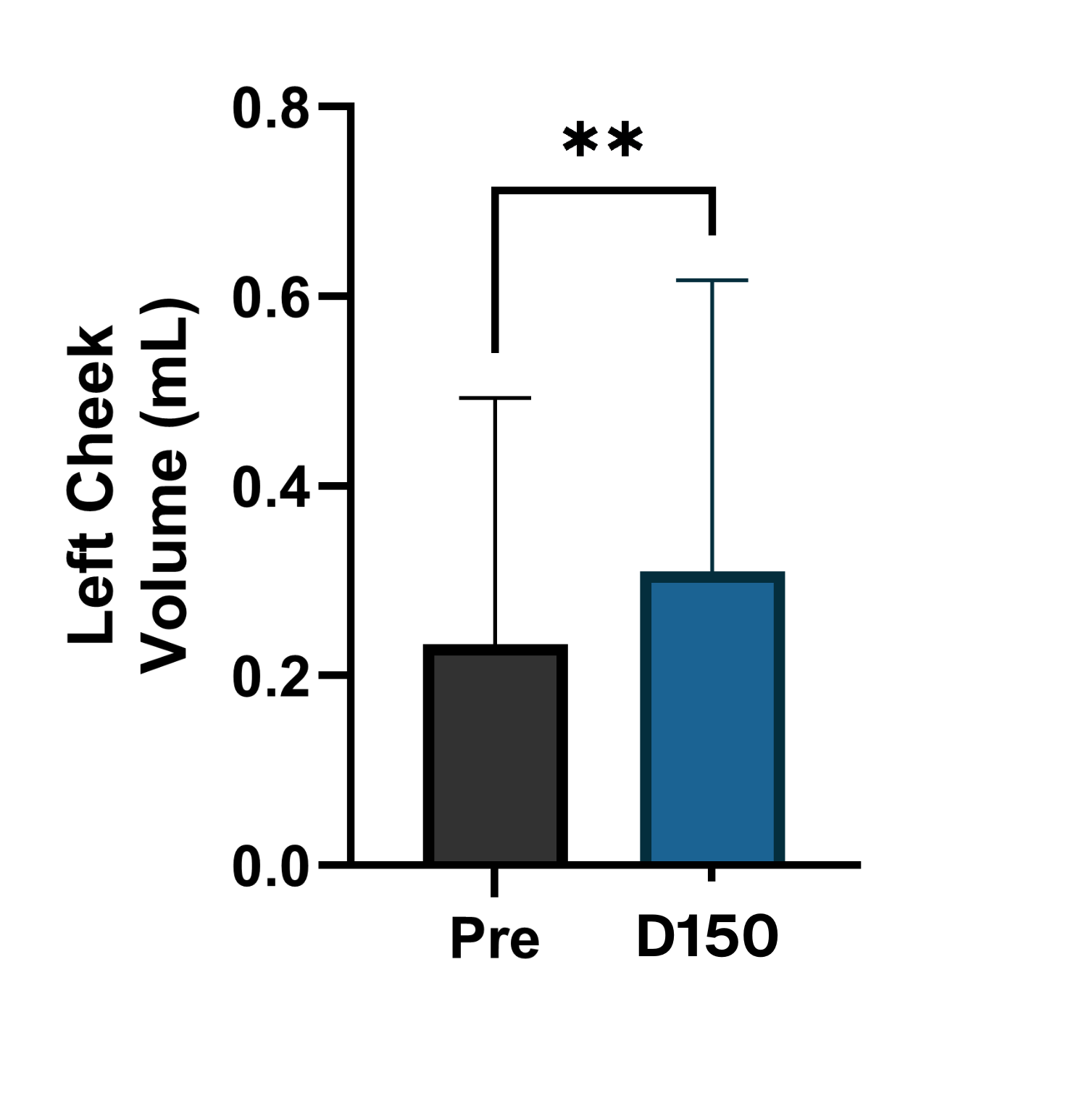

Supplement: ojaf104_Supplementary_Data [file ojaf104_Supplementary_Data.zip › Supplemental Figure 1A.png]

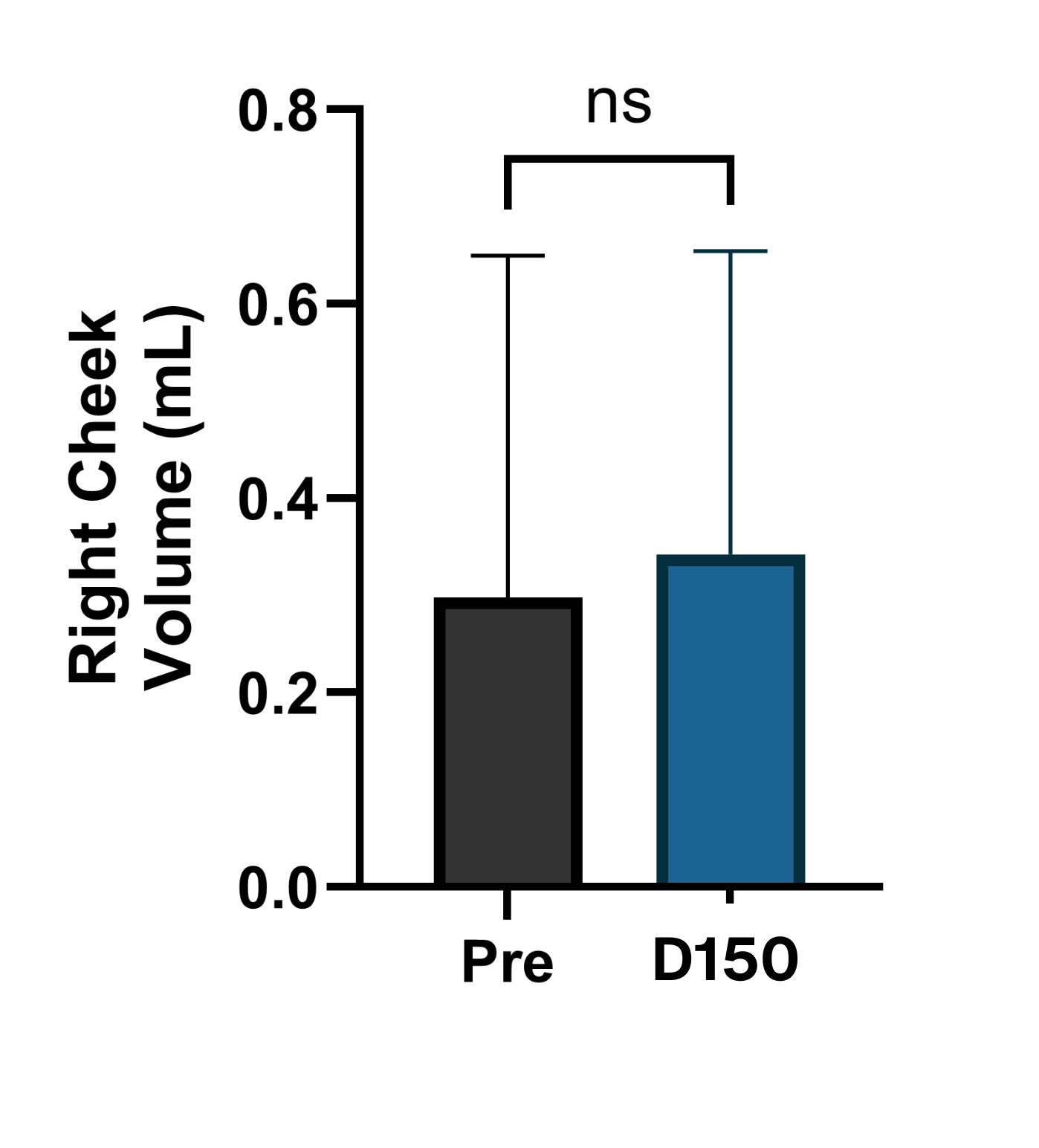

Supplement: ojaf104_Supplementary_Data [file ojaf104_Supplementary_Data.zip › Supplemental Figure 1B.png]

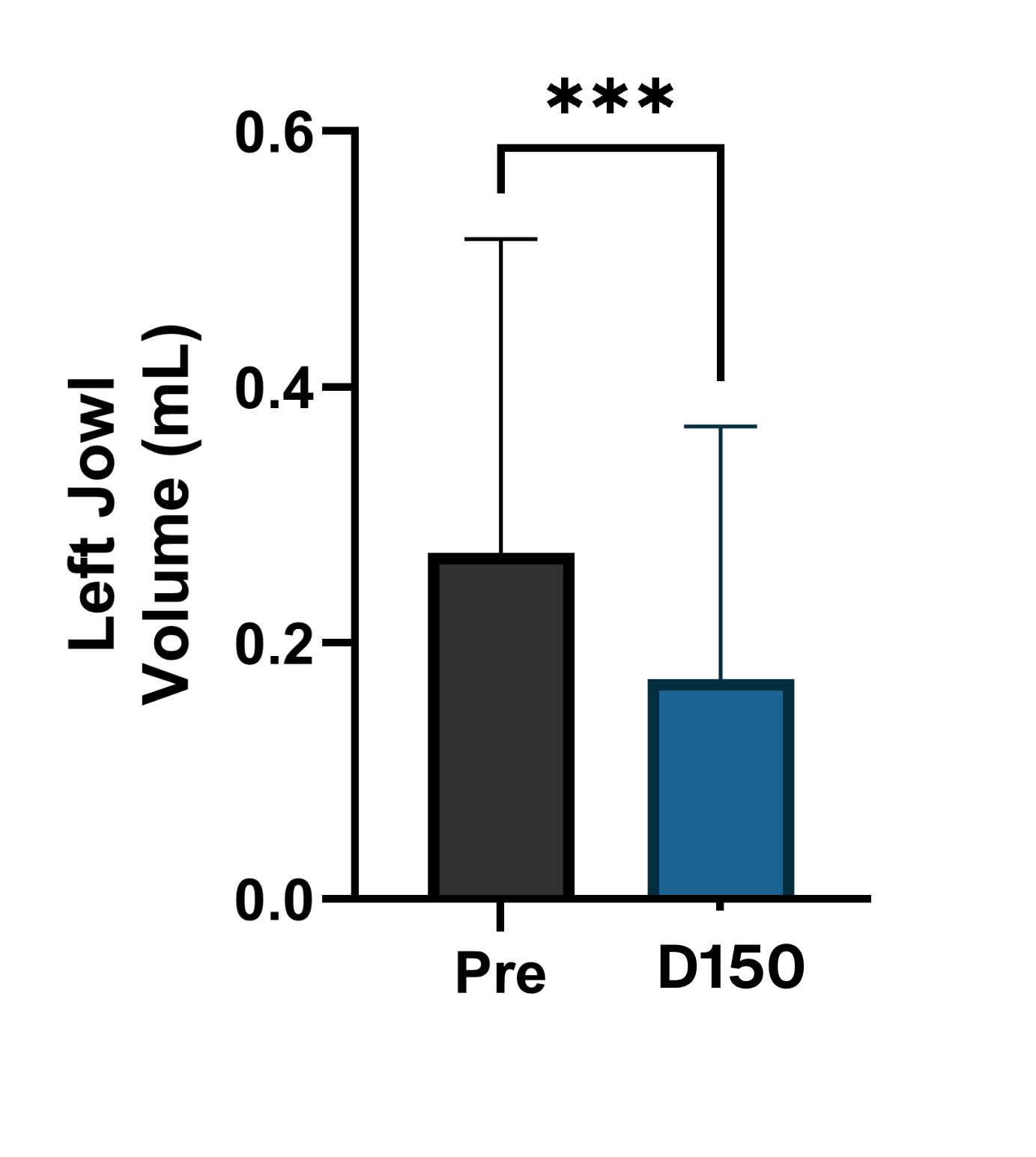

Supplement: ojaf104_Supplementary_Data [file ojaf104_Supplementary_Data.zip › Supplemental Figure 2A.png]

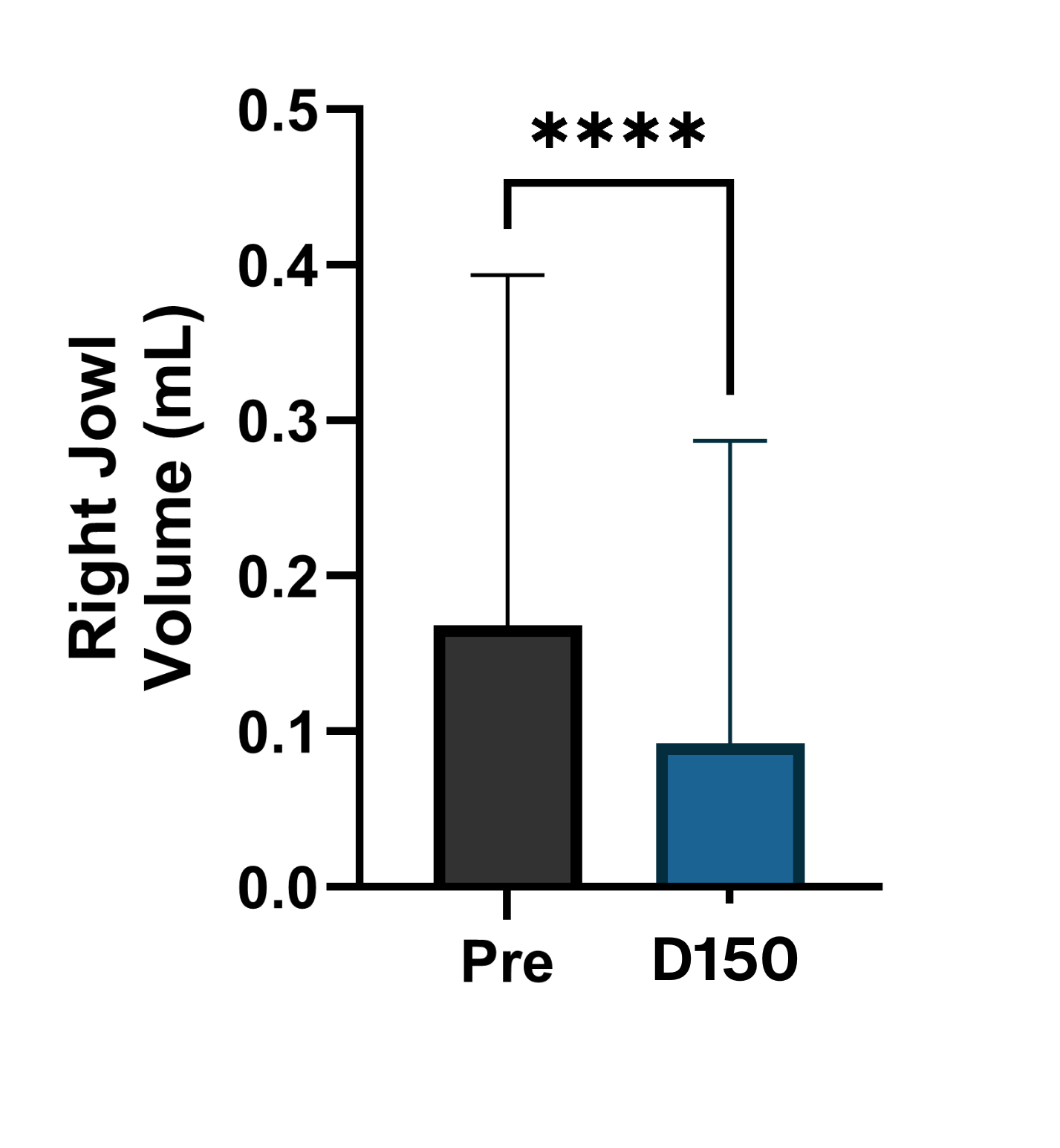

Supplement: ojaf104_Supplementary_Data [file ojaf104_Supplementary_Data.zip › Supplemental Figure 2B.png]

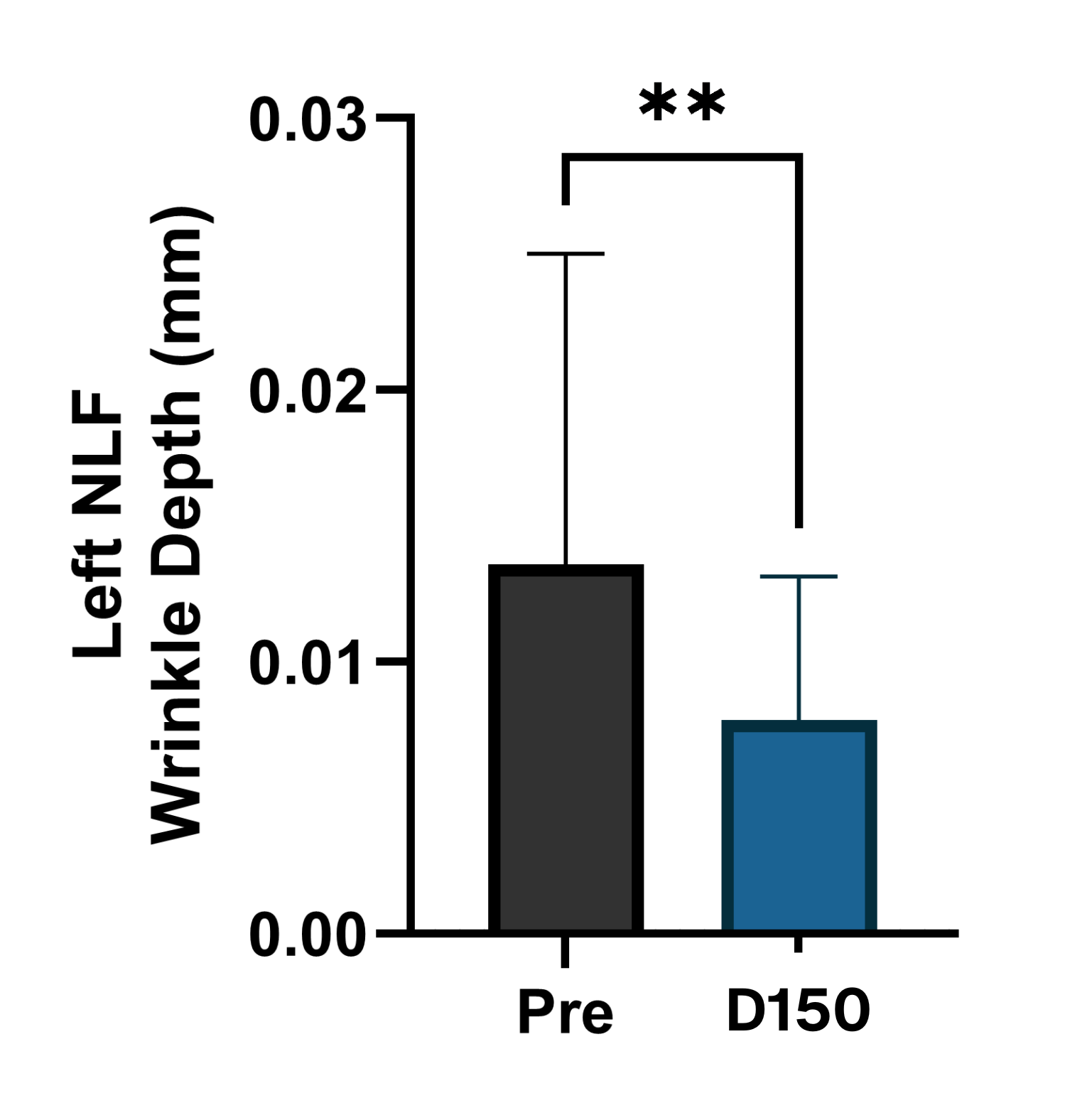

Supplement: ojaf104_Supplementary_Data [file ojaf104_Supplementary_Data.zip › Supplemental Figure 3A.png]

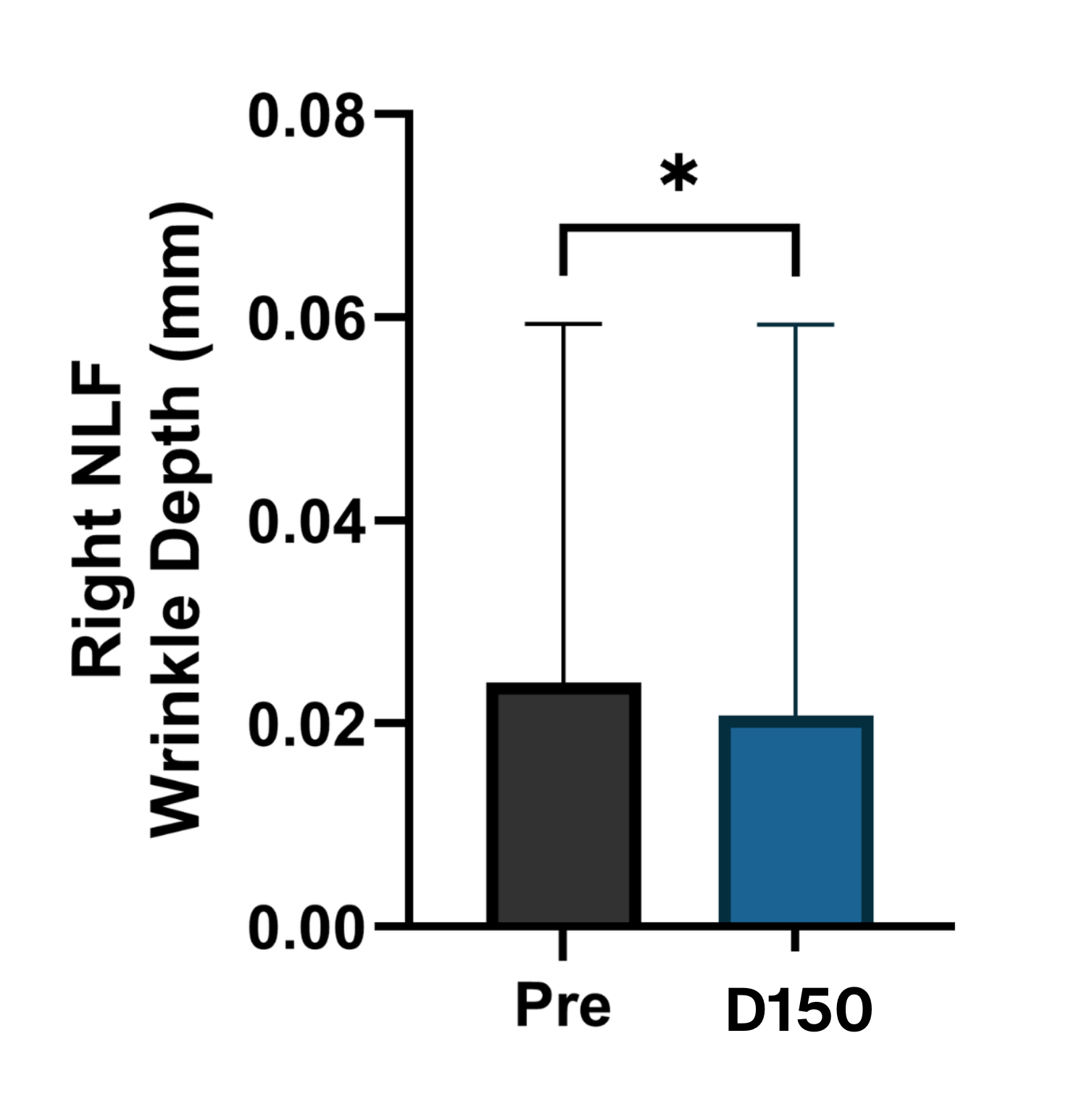

Supplement: ojaf104_Supplementary_Data [file ojaf104_Supplementary_Data.zip › Supplemental Figure 3B.png]

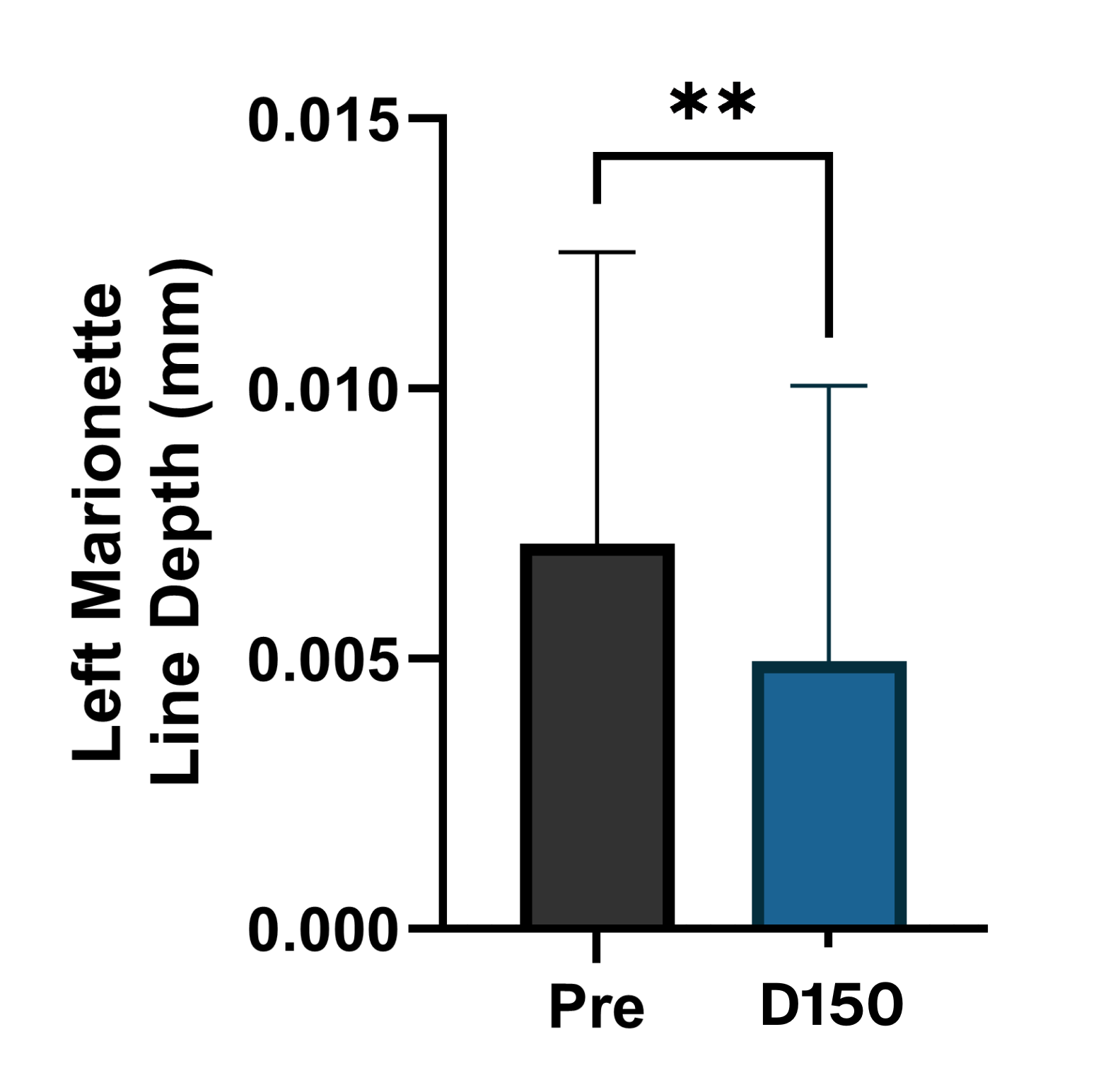

Supplement: ojaf104_Supplementary_Data [file ojaf104_Supplementary_Data.zip › Supplemental Figure 4A.png]

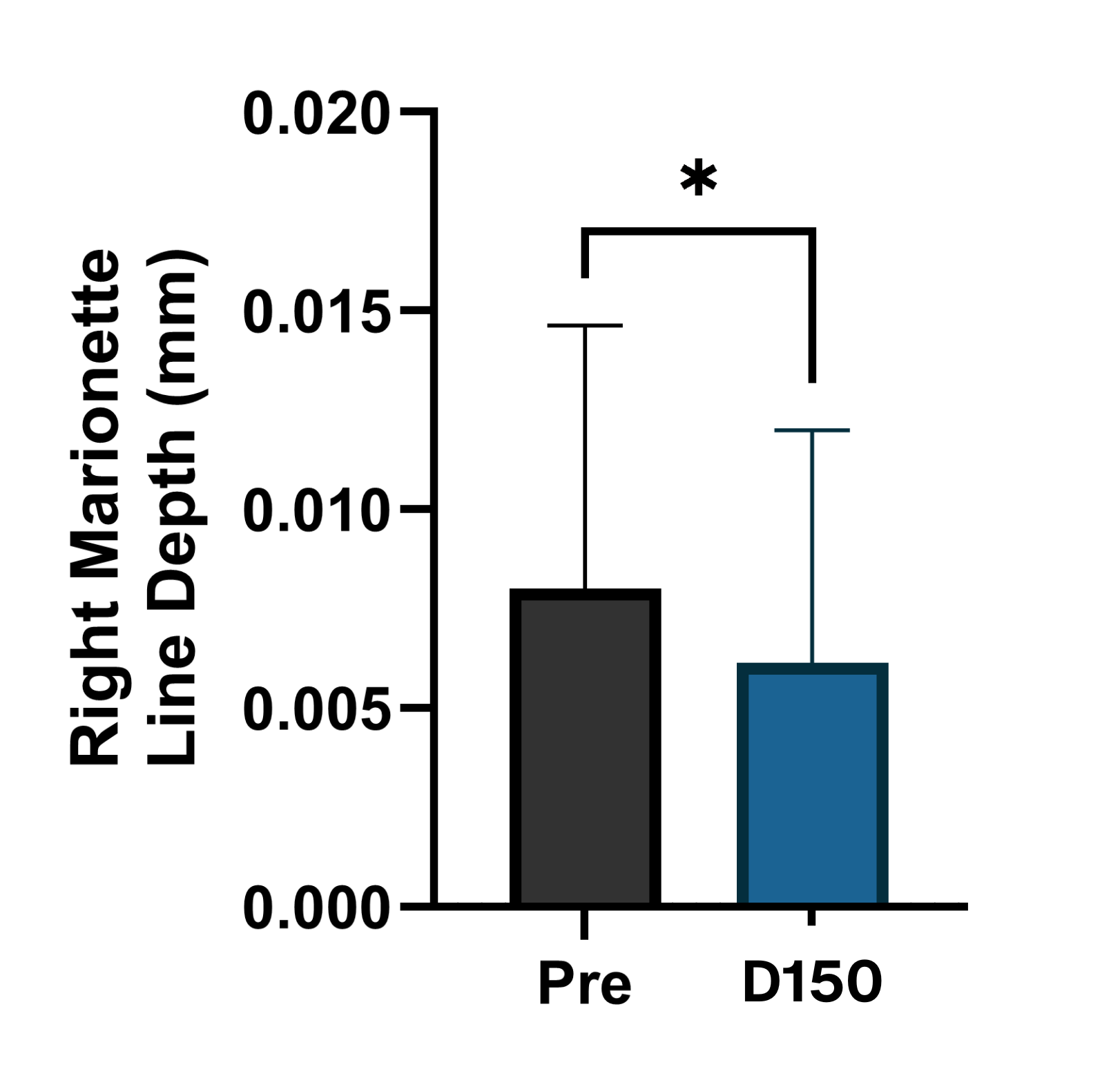

Supplement: ojaf104_Supplementary_Data [file ojaf104_Supplementary_Data.zip › Supplemental Figure 4B.png]

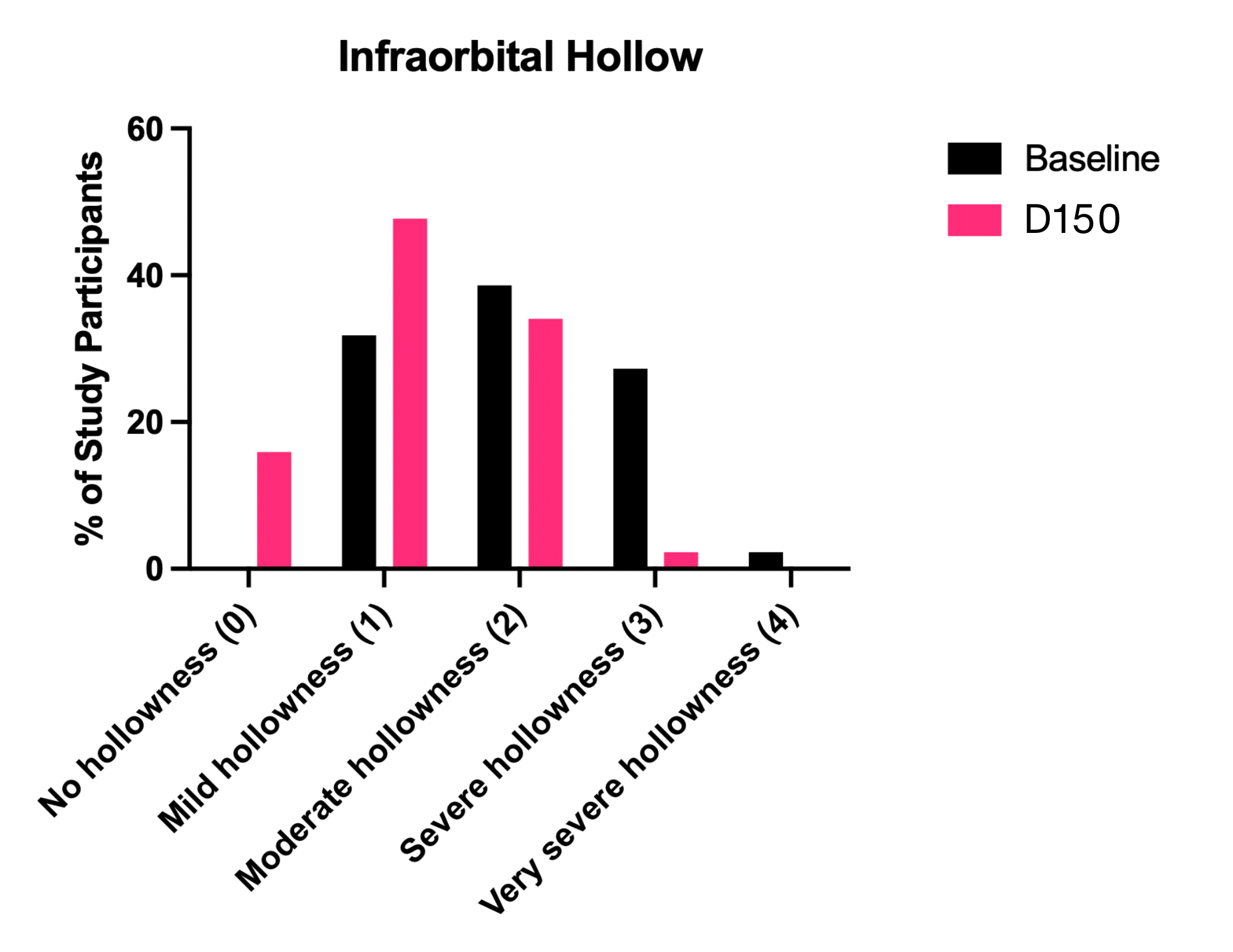

Supplement: ojaf104_Supplementary_Data [file ojaf104_Supplementary_Data.zip › Supplemental Figure 5A.png]

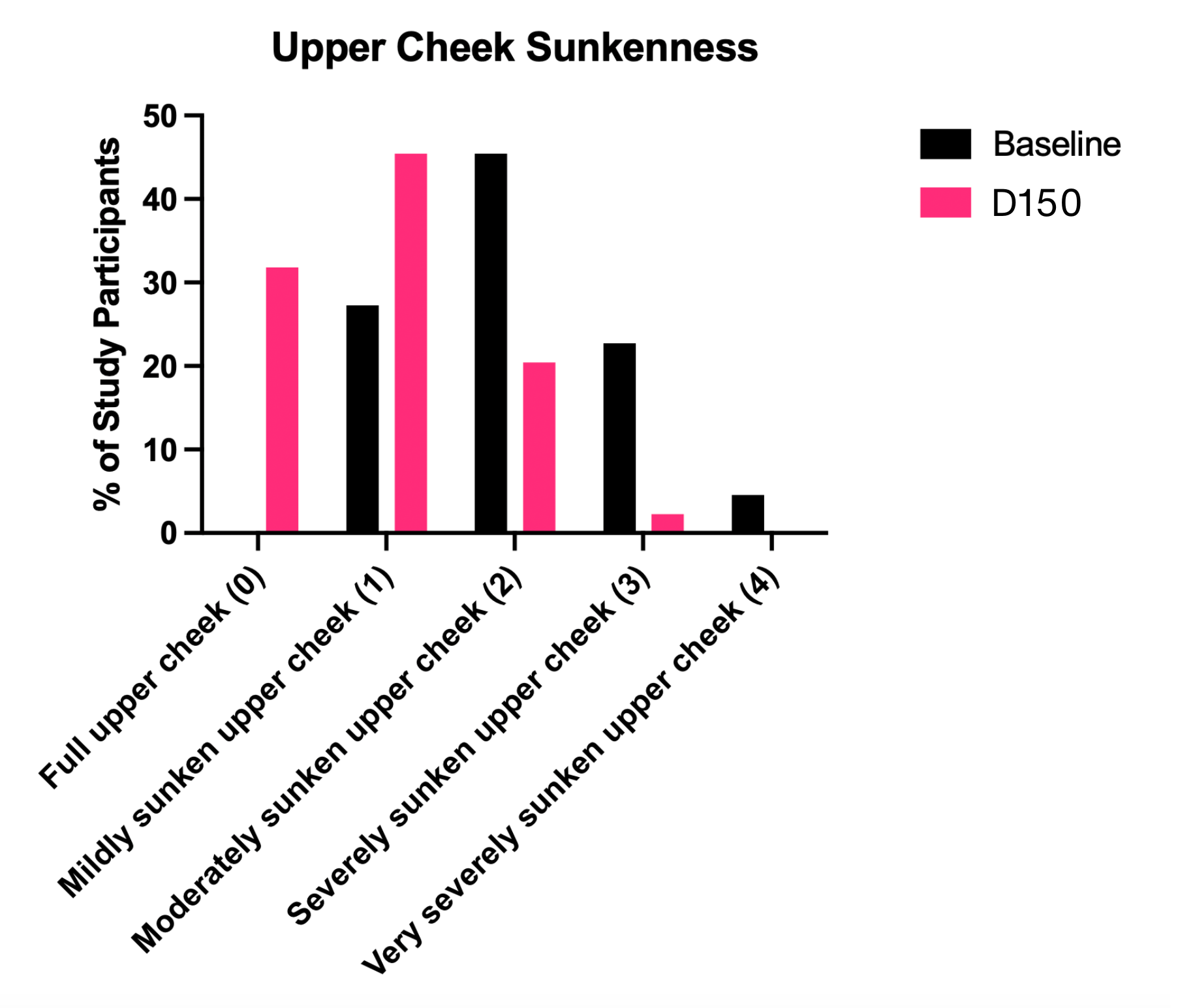

Supplement: ojaf104_Supplementary_Data [file ojaf104_Supplementary_Data.zip › Supplemental Figure 5B.png]

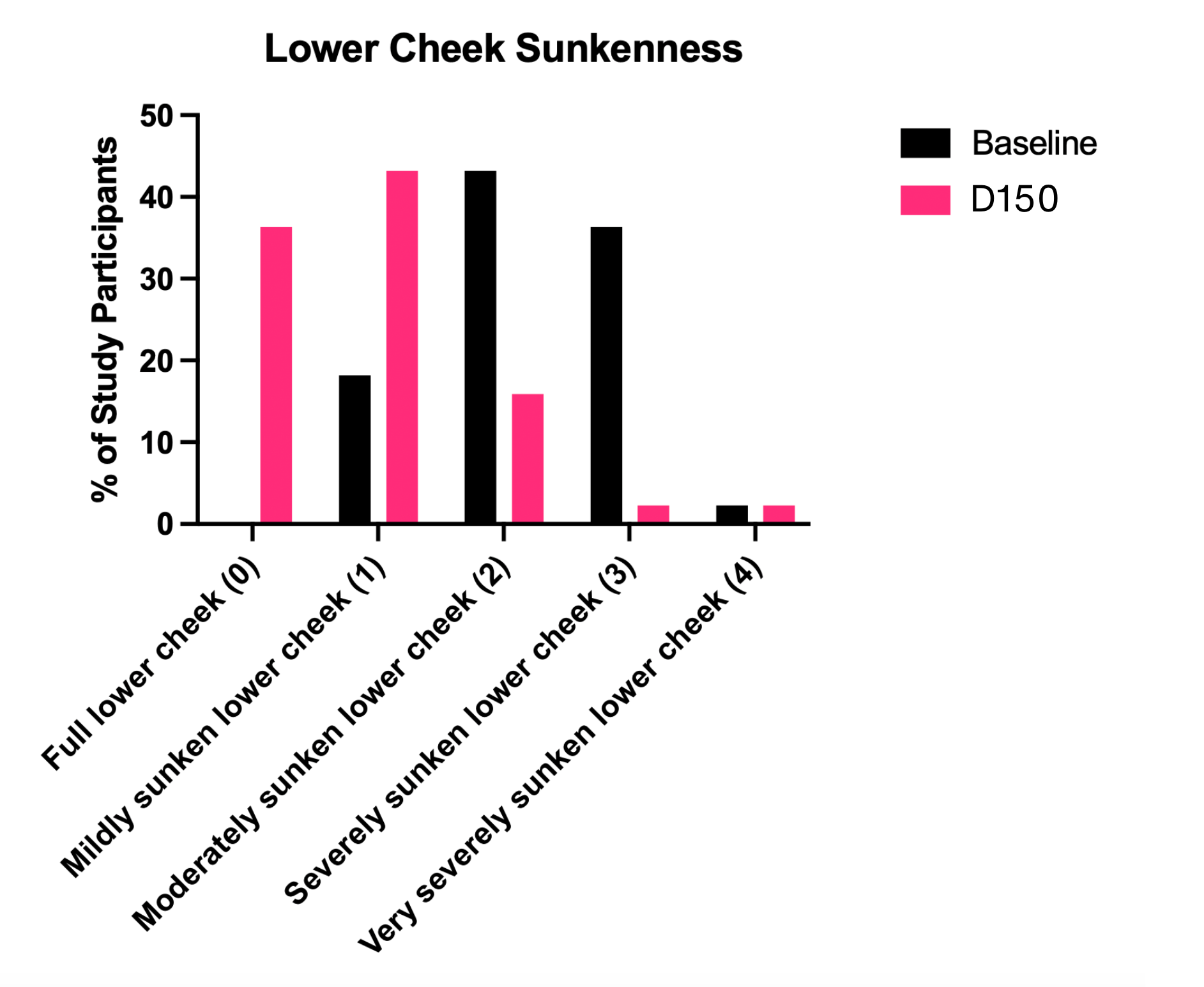

Supplement: ojaf104_Supplementary_Data [file ojaf104_Supplementary_Data.zip › Supplemental Figure 5C.png]

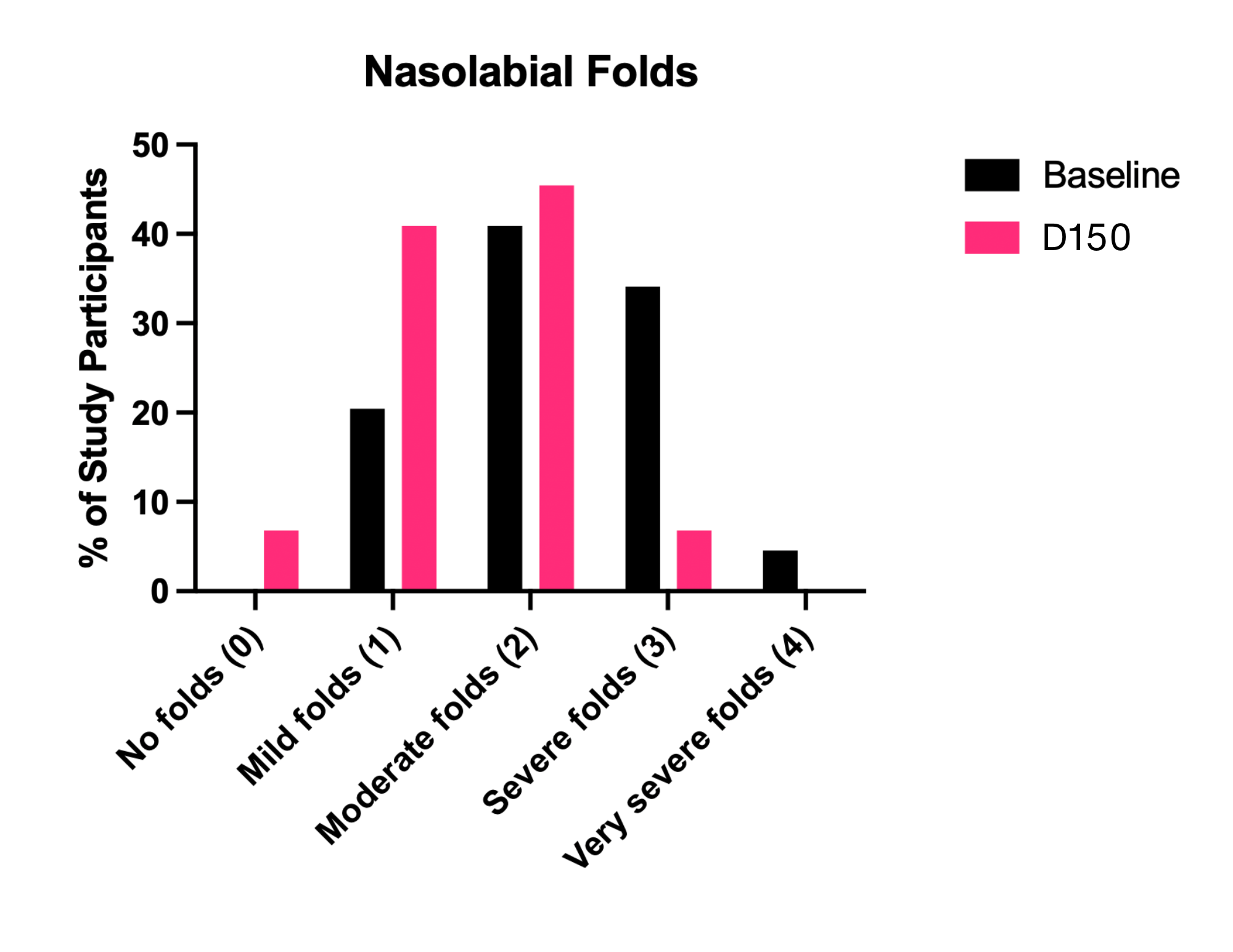

Supplement: ojaf104_Supplementary_Data [file ojaf104_Supplementary_Data.zip › Supplemental Figure 5D.png]

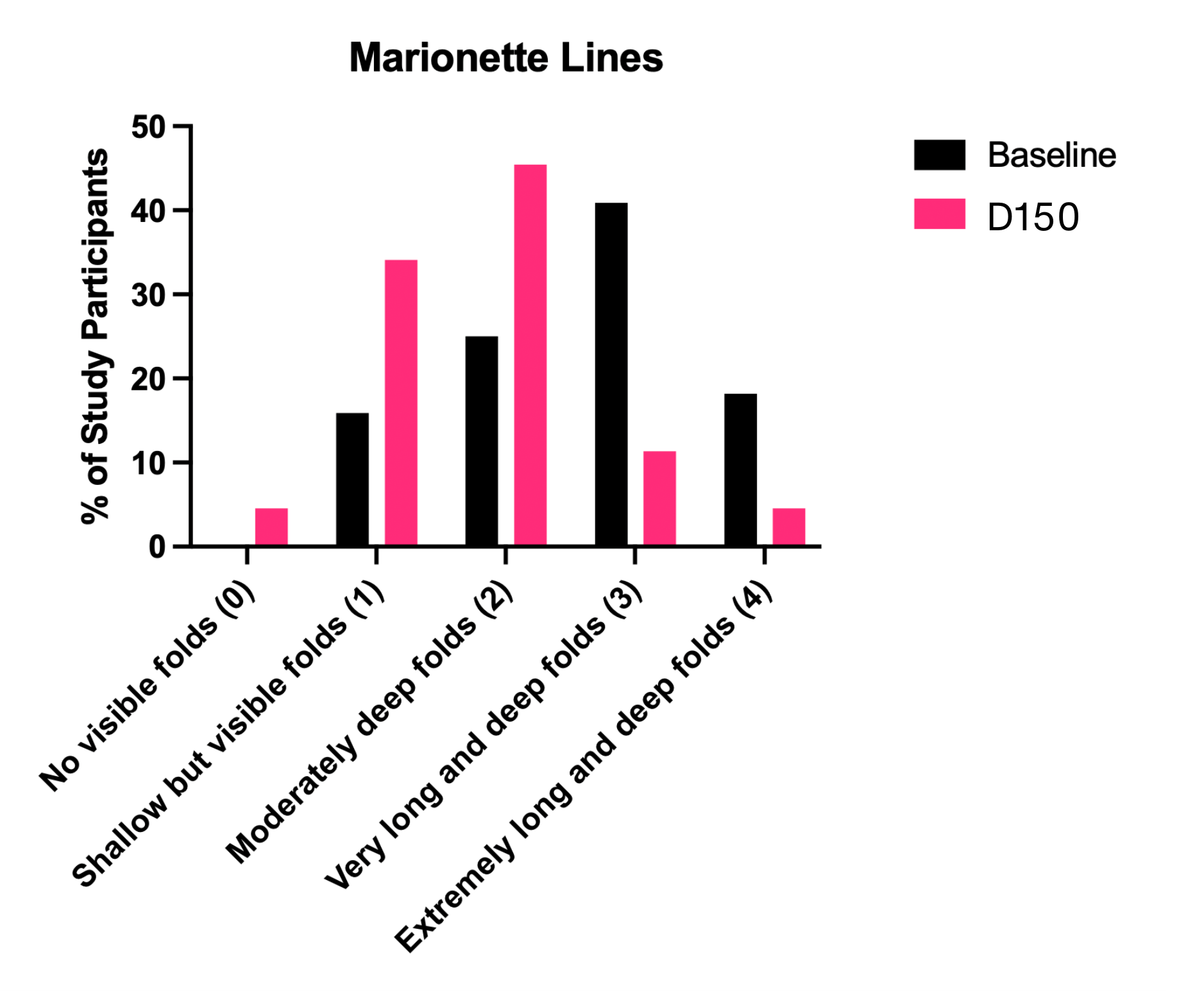

Supplement: ojaf104_Supplementary_Data [file ojaf104_Supplementary_Data.zip › Supplemental Figure 5E.png]

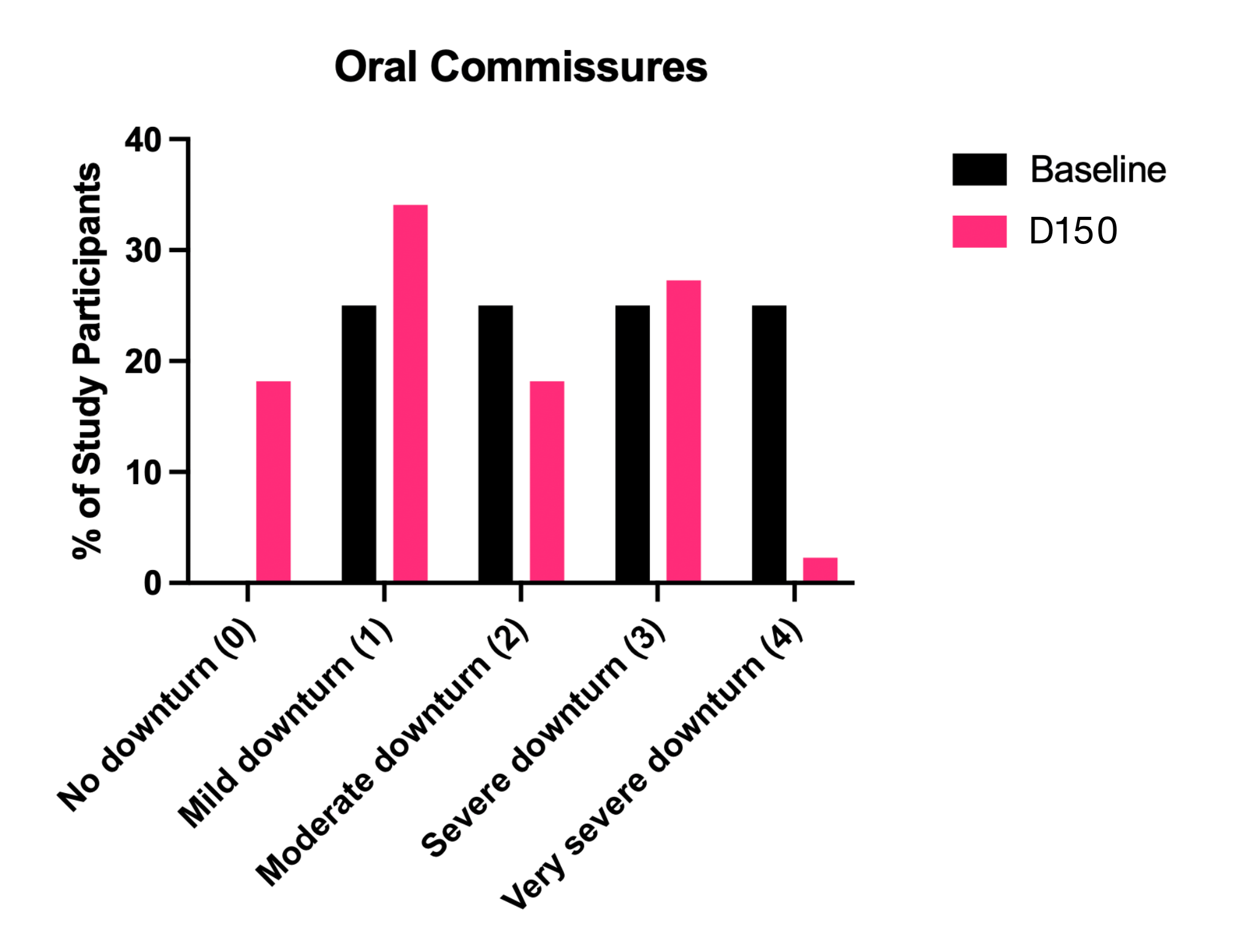

Supplement: ojaf104_Supplementary_Data [file ojaf104_Supplementary_Data.zip › Supplemental Figure 5F.png]

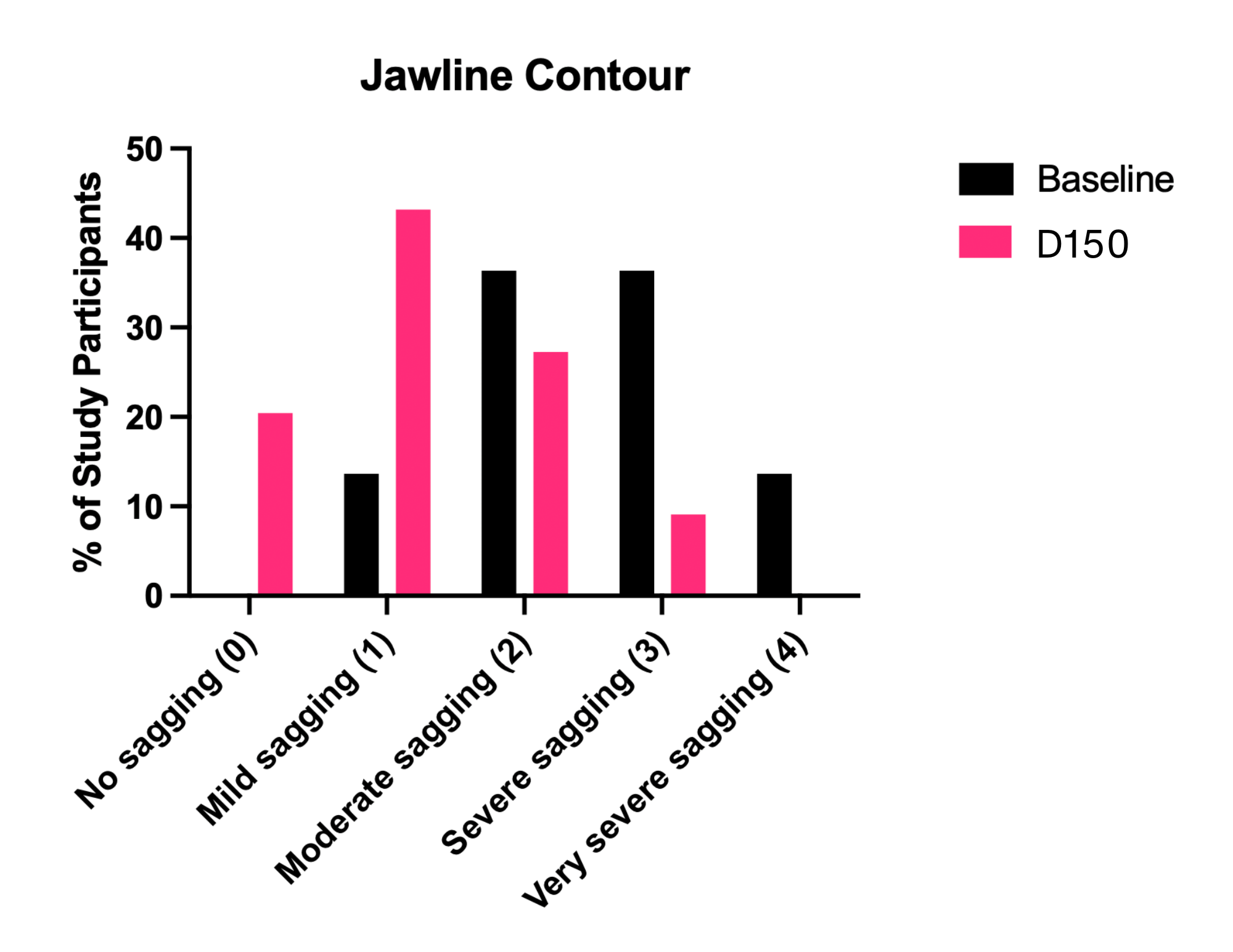

Supplement: ojaf104_Supplementary_Data [file ojaf104_Supplementary_Data.zip › Supplemental Figure 5G.png]

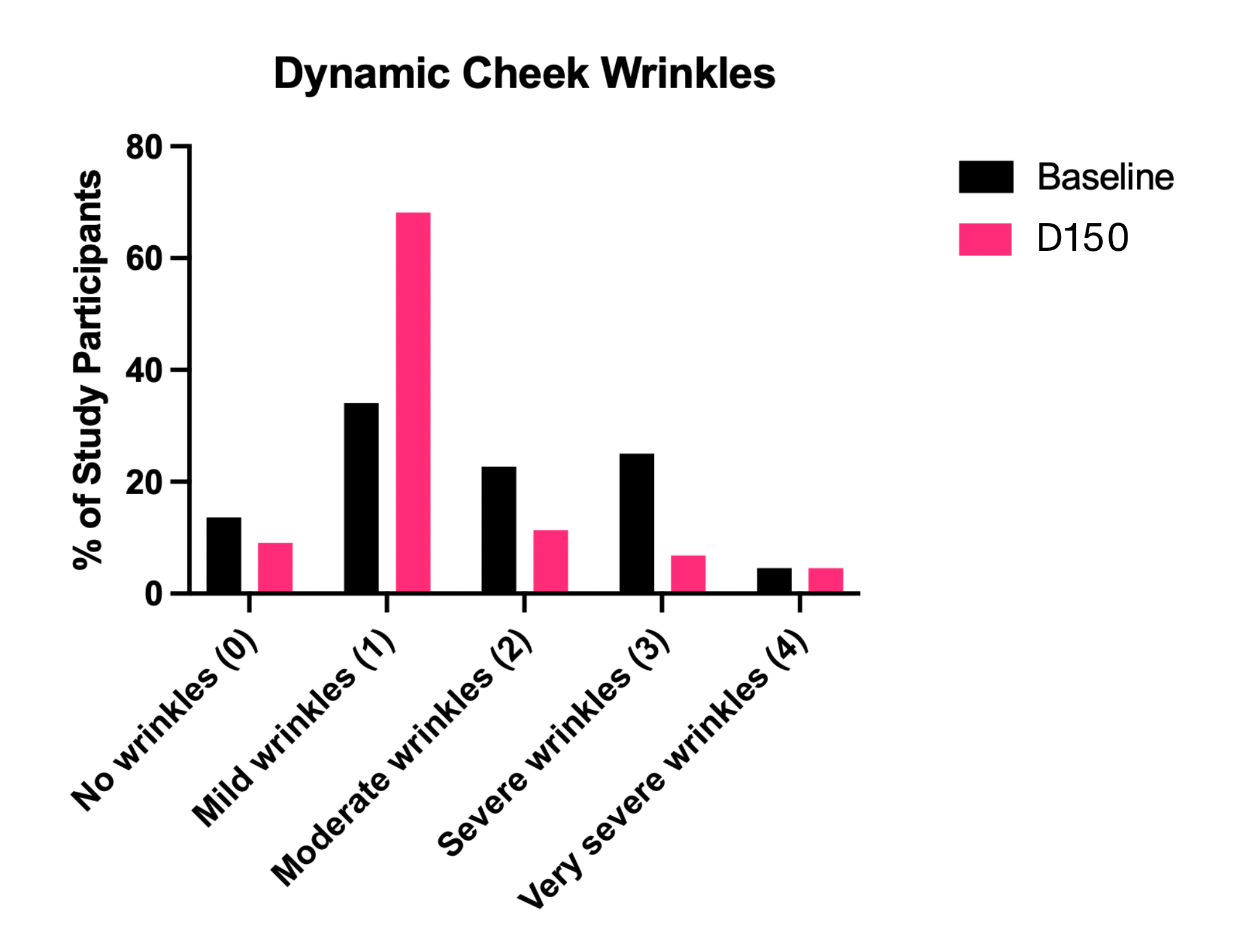

Supplement: ojaf104_Supplementary_Data [file ojaf104_Supplementary_Data.zip › Supplemental Figure 5H.png]

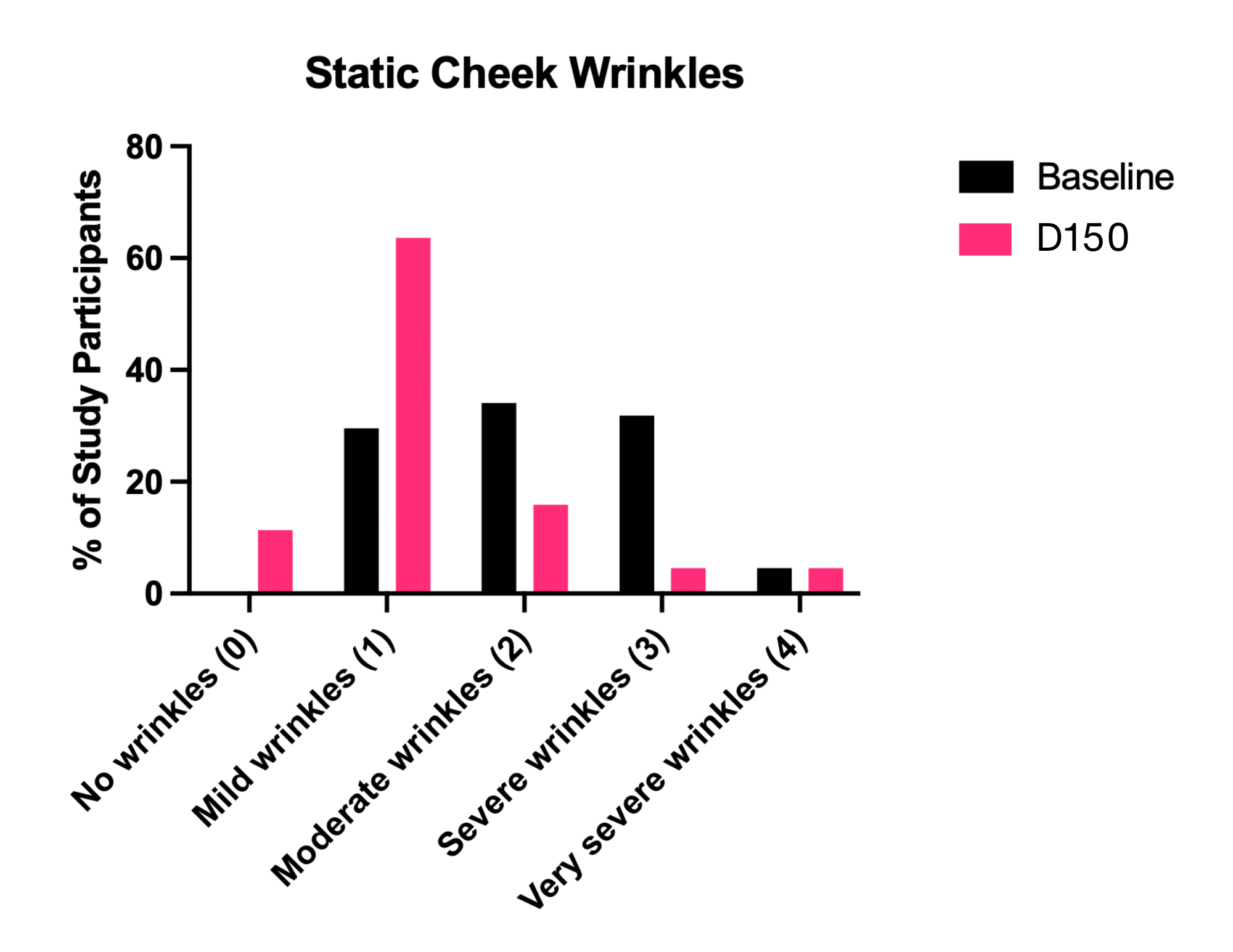

Supplement: ojaf104_Supplementary_Data [file ojaf104_Supplementary_Data.zip › Supplemental Figure 5I.png]
